# Turmeric extract-mediated biogenic synthesis of Ag@SeO_2_ magnetic nanoparticles: characterization, optimization, antibacterial and antioxidant activities†

**DOI:** 10.1039/d4ra00004h

**Published:** 2024-02-27

**Authors:** Abeer A. Ghoniem, Khaled M. Elattar, Fatimah O. Al-Otibi, Ashraf Elsayed, Mohammed S. El-Hersh, Ayman Y. El-Khateeb, Yosra A. Helmy, WesamEldin I. A. Saber

**Affiliations:** a Microbial Activity Unit, Department of Microbiology, Soils, Water and Environment Research Institute, Agricultural Research Center Giza 12619 Egypt abeer.abdelkhalik@yahoo.com m.elhersh@yahoo.com wesameldin.saber@arc.sci.eg; b Unit of Genetic Engineering and Biotechnology, Faculty of Science, Mansoura University El-Gomhoria St. Mansoura 35516 Egypt khaledelattar2@yahoo.com; c Botany and Microbiology Department, Faculty of Science, King Saud University Riyadh 11451 Saudi Arabia falotibi@ksu.edu.sa; d Botany Department, Faculty of Science, Mansoura University El-Gomhoria St. Mansoura 35516 Egypt ashraf-badawy@mans.edu.eg; e Agricultural Chemistry Department, Faculty of Agriculture, Mansoura University El-Gomhoria St. Mansoura 35516 Egypt aymanco@mans.edu.eg; f Department of Veterinary Science, Martin-Gatton College of Agriculture, Food, and Environment, University of Kentucky Lexington KY 40546 USA yosra.helmy@uky.edu

## Abstract

This study bio-synthesized Ag@SeO_2_ bmNPs successfully, using turmeric ethanol extract, and characterized them using various techniques. The FT-IR analysis reveals the involvement of these plant-derived compounds, especially phenolics, in the reduction process by acting as electron donors and stabilizing/capping agents. Zeta potential analysis showed a slight negative surface charge for the stability of Ag@SeO_2_ NPs, where TEM revealed spherical nanoparticles with an average size of 20 nm. The XRD confirmed crystallinity and a core–shell structure, and EDX identified elements consistent with Ag@SeO_2_ and a 3 : 1 Ag/Se atomic ratio. Further, SEM supported the spherical shape and uniform size. These findings highlight the successful biosynthesis of Ag@SeO_2_ bmNPs with promising properties for diverse applications. Moreover, the Box–Behnken design (BBD) and artificial neural network (ANN) model were engaged to optimize Ag@SeO_2_ bmNP biosynthesis. BBD identified significant influences of pH, bioconversion temperature, time, and turmeric concentration on bmNP yield, with adjusted *R*^2^ and predictive *R*^2^ being 0.9075 and 0.8829, respectively. However, its limitations were revealed by a significant lack of fit. ANN modeling with a 3–5–7–1 topology showed superior predictive accuracy and identified optimal conditions for maximizing yield (pH 9.83, 51.7 °C, 1.0 h, 3.71 mg mL^−1^ turmeric). Validation experiments confirmed the model's reliability. Turmeric extract exhibited significantly higher amounts of phenolics, and flavonoids compared to the bmNPs, suggesting its potential for strong antioxidant activity. Both turmeric extract and bmNPs displayed antioxidant activity in ABTS and DPPH assays, with turmeric extract being the most potent due to its curcuminoid content. The potential activity of Ag@SeO_2_ bmNPs against *S. aureus*, *K. pneumonia*, *E. coli*, and *B. cereus* was investigated, with inhibition zones ranging from 22 to 32 mm. The MIC values of tested NPs towards pathogenic bacteria ranged from 165.625 and 331.25 μg mL^−1^.

## Abstract 2

Biosynthesis of Ag@SeO_2_ NC using turmeric extract with promising antibacterial and antioxidant properties, optimized using AI, and confirmed by various techniques.

## Graphical abstract

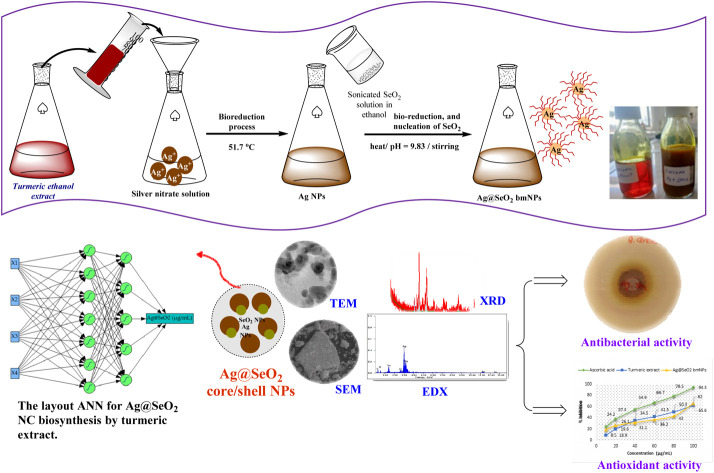

## 1. Introduction

Biogenic nanoparticles (NPs) are nanoparticles that are synthesized using biological resources, such as plants, bacteria, and fungi. Biogenic NPs have several advantages over chemically synthesized NPs, including their low cost, ease of synthesis, and biocompatibility.^1,2^ Turmeric extract is a rich source of curcumin, a bioactive compound with a wide range of biological activities, including antioxidant, anti-inflammatory, and anticancer activities.^3–6^ Curcumin has also been shown to reduce the toxicity of silver nanoparticles (Ag-NPs) as well as the expected improved antibacterial activity owing to the synergistic effects of Ag, Se, and curcumin.^7^

Selenium dioxide (SeO_2_) is a semiconductor material with a wide range of applications in catalysis, electronics, and biomedicine.^8^ SeO_2_ NPs have been shown to exhibit excellent antifungal and antioxidant activities.^9,10^ The bmNPs have the potential to be used in a variety of applications, including biomedical applications for developing new drugs and diagnostic tools for the treatment and detection of fungal infections and other diseases.^11^ The bmNPs could be used in the development of new water purification and wastewater treatment technologies.^12^ The bmNPs could be used in the development of new food packaging materials and antimicrobial coatings to prevent food spoilage.^13^

Plant-mediated synthesis of metal nanoparticles (PMSN) is a green and sustainable approach to producing metal nanoparticles using plant extracts. It has emerged as a promising alternative to traditional physical and chemical methods, which are often energy-intensive, expensive, and environmentally unfavorable.^14^ PMSN harnesses the unique properties of plant extracts to reduce and stabilize metal ions into nanoparticles. Plant extracts contain a variety of phytochemicals, such as alkaloids, flavonoids, terpenoids, and polysaccharides, which can act as reducing agents, capping agents, and stabilizers.^15,16^ These phytochemicals can also control the size, shape, and properties of the synthesized nanoparticles.

The optimization of metallic nanoparticle synthesis is a critical facet involving the meticulous identification and adjustment of reaction parameters to achieve nanoparticles possessing the desired size, shape, and properties.^17^ Diverse factors exert influence throughout this process; notably, precursor salt concentration plays a pivotal role, impacting both nanoparticle size and nucleation rate.^18^ The nature and concentration of the reducing agent are significant determinants affecting the resulting size, shape, and properties of the nanoparticles, whereas capping agents serve the crucial role of stabilizing nanoparticles and preventing aggregation.^19,20^ Reaction temperature modulates nucleation and growth rates, while pH exerts influence over precursor salt solubility and nanoparticle stability.^21^ Although one-at-a-time optimization, albeit straightforward, proves time-intensive and may not ascertain the true optimum,^22^ the experimental design approach of response surface methodology permits the simultaneous statistical analysis of multiple parameters, revealing intricate interactions.^23^ Furthermore, the integration of machine learning facilitates the prediction of nanoparticle properties based on synthesis parameters, thereby guiding the optimization process.^24^ The refinement of synthesis is exemplified by the controlled modulation of the size and nucleation rate of silver nanoparticles through the adjustment of precursor salt concentration.^25^ Recognizing the pivotal role of optimization in tailoring metallic nanoparticles with specific characteristics for diverse applications, scientists rigorously control synthesis conditions to ensure precise outcomes.^26,27^

In the burgeoning field of nanotechnology, biological researchers are trying to unveil a pioneering application of artificial intelligence in the modeling of biosynthesis for nanometals, and leveraging the unique capacity of artificial neural networks (ANNs) to capture temporal dependencies and intricate sequential patterns. Our study presents a methodological framework that brings unprecedented insight into the dynamic molecular processes involved.

The advantages of employing ANNs become evident as they outperform traditional modeling approaches. The benefits of employing ANNs range from their ability to discern complex biosynthetic pathways to predicting optimal conditions for enhanced efficiency. The iterative nature of ANNs mirrors the iterative intricacies of biosynthesis, enabling a more nuanced and precise representation of the intricate interplay of biological components.^28,29^ This research sheds light on the seamless integration of ANNs as a transformative tool, offering a data-driven lens that not only elucidates the biosynthesis of nonmetals but also opens new avenues for optimizing and advancing nanometal production.

Turmeric ethanol extract is a popular herbal supplement that is made by soaking turmeric rhizomes in ethanol. Ethanol is a good solvent for extracting active phytochemicals from turmeric, including curcuminoids, volatile oils, and other phenolic compounds.^30^ Curcuminoids are the most active phytochemical components of turmeric ethanol extract.^31^ Curcuminoids are polyphenolic compounds that have been shown to have a widespread variety of biological activities, comprising antioxidant, anti-inflammatory, and anticancer features.^32^ The three main curcuminoids are curcumin, demethoxy-curcumin, and *bis*-demethoxy-curcumin.^33^ Curcumin is the most abundant and well-studied curcuminoid.^34^ Volatile oils are another important class of phytochemicals in turmeric ethanol extract. Volatile oils are liable for the pungent aroma of turmeric.^35^

The main volatile oils in turmeric are turmerone, ar-turmerone, and zingiberene. Volatile oils have been shown to have antibacterial, antifungal, and insecticidal properties.^36^ Other phenolic compounds in turmeric ethanol extract include ar-curcumin, ferulic acid, and caffeic acid. These compounds also displayed a variety of biological features, including antioxidant, anti-inflammatory, and anticancer features.^37^ Turmeric ethanol extract presents a wide assortment of potential health benefits as it is considered to reduce inflammation, boost the immune system, protect against heart disease, prevent cancer, improve brain function, relieve pain and arthritis symptoms, protect the liver, and improve digestion.^38,39^ Turmeric ethanol extract is generally safe for most people to take. Turmeric extracts protect the brain from damage caused by neurodegenerative diseases such as Alzheimer's disease and Parkinson's disease.^40^ Turmeric extracts have also been shown to protect the heart from damage caused by heart disease. This is due to their ability to improve cholesterol levels, reduce inflammation, and prevent blood clots.^41^ In addition to these biological activities, turmeric extracts have also been shown to have beneficial effects on digestion, liver health, and mood.^42^

In this study, we report the green synthesis and characterization of Ag–SeO_2_ core/shell magnetic nanoparticles using turmeric extract as a reducing and stabilizing agent. We also investigate the optimization of the synthesis process, phytochemical analyses, and the antibacterial and antioxidant activities of the turmeric extract and Ag–SeO_2_ bmNPs.

## 2. Materials and methods

### 2.1. Instruments

Fourier-transform infrared spectroscopy (FT-IR) analysis was performed on a Thermo-Fisher Nicolet IS10 Spectrophotometer in the frequency range of 500–4000 cm^−1^. Zetasizer and zeta potential analyses were performed on HORIBA Scientific SZ-100 “Ver 2.40”. The surface features, shape, and elemental composition of the silver-selenium dioxide nanocomposite were analyzed using scanning electron microscopy (SEM) and energy dispersive X-ray spectroscopy (EDX) on an FEI Czech SEM-type instrument at an accelerating voltage of 25 kV. Transmission electron microscopy (TEM) analyses were performed on a ThermoScientific Talos F200i instrument using a carbon-coated grid. X-ray diffraction (XRD) analysis was performed on a Pan Analytical Philips instrument to determine the material type, phase, crystallographic structure, and physical properties of the nanocomposite.

### 2.2. Preparation of turmeric extract

Turmeric (*Curcuma longa*) powder was purchased from the local market in Mansoura city, Egypt. A weighed 10 grams of turmeric powder was placed in a clean conical flask (250 mL). 100 mL of 80% ethanol was added to the plant powder and the mixture was stirred to ensure that all of the turmeric powder was wetted by the ethanol. The flask was covered with a lid or parafilm and placed in a dark at 25 °C. The mixture was soaked for 24 hours and stirred occasionally.^43^ The mixture was then filtered with a filter paper and the filtrate was used immediately. The turmeric extract was stored in a cool, and dark place to preserve its quality.

### 2.3. Biosynthesis and storage of Ag@SeO_2_ nanocomposite

#### 2.3.1. Biosynthesis

A solution of silver nitrate (1 mM) (AgNO_3_: PIOCHEM for laboratory chemicals; CAS number: 7761-88-8; purity: 99.5%) was prepared in distilled water. A suspension of selenium dioxide was prepared in deionized water (1 mM). Turmeric extract (20 mL, 3.71 mg mL^−1^) was added dropwise to the AgNO_3_ solution (10 mL, 1 mM). The extract acted as a reducing agent and reduced the silver ions (Ag^+^) to silver nanoparticles (Ag-mNPs). The mixture was stirred at 51.7 °C until the color of the solution changed to brown. The selenium dioxide suspension (10 mL, 1 mM) (SeO_2_: Alfa Aesar, Thermo Fisher (Kandel) GmbH, Erienbachweg 2, Germany, purity: 99.4%, CAS number: 7446-08-4) was added to the mixture of the AgNO_3_ solution and the turmeric extract. The pH was adjusted to 9.83 using sodium hydroxide solution (1 M), in which the solution pH was maintained by a pH meter. The mixture was stirred for 1 hour to allow the Ag@SeO_2_ nanocomposite to form completely. The mixture was centrifuged to collect the Ag@SeO_2_ nanocomposite. The formed nanocomposite was washed with ethanol and deionized water to remove any impurities and contaminated oil. The Ag@SeO_2_ nanocomposite was dried at 60 °C for 24 hours.^44^

#### 2.3.2. Storage conditions

Experiments were conducted on storage stability under the recommended conditions for a month to confirm their stability. Temperature: the sample was stored between 4 and 8 °C (refrigerator). Higher temperatures can accelerate the degradation of both the nanoparticles and capping agents. Humidity: Low humidity (around 30–40%) should be maintained to prevent moisture-induced aggregation. Desiccators or airtight containers should be considered with desiccants. Light exposure: the sample should be stored in the dark as light can induce degradation in some nanoparticles and can affect the capping agents as well. Amber or opaque containers should be used. Storage container: glass or quartz containers are preferred to avoid leaching. pH: it's crucial to measure and adjust the final pH of the stored nanoparticles for optimal stability. Aim for a slightly acidic pH (around 5–6) as this can improve the stability of both the nanoparticles and capping agents. Dispersing medium: the nanoparticles should be resuspended in a suitable solvent or buffer before storage. Physiological buffers with moderate ionic strength are often preferred to maintain the integrity of the capping agents. Harsh organic solvents should be avoided. Monitoring: the nanoparticles should be regularly monitored for changes in size, zeta potential, and visual appearance during storage to detect any instability issues. Any signs of aggregation or degradation of the capping agents should be paid attention.

### 2.4. Optimization Ag@SeO_2_ biosynthesis conditions

#### 2.4.1. Box–Behnken design (BBD)

The experimental matrix of the BBD was constructed to model the optimum operation conditions that maximize the green synthesis of Ag@SeO_2_ bmNPs by turmeric extract. Various experimental combinations were designed and then experimentally performed, and the Ag@SeO_2_ bmNPs, as a response, were measured. On this connection, the combined action of four variables, including pH, bioconversion temperature (°C), bioconversion time (h), and concentration of turmeric extract (mg mL^−1^), were tested for maximization of the bioconversion process. The objective was to find out the best combination of the four independent variables thus the optimum Ag@SeO_2_ bmNPs biosynthesis could be determined.

According to BBD, each of the four factors was examined at low, middle, and high levels. Thus, a four-factor matrix of various combinations of experimental runs was generated, composed of 27 runs as shown in Table 1. The design was experimentally repeated to ensure accuracy, and each run was carried out in triplicates. Once performing the laboratory experiments, the biosynthesized Ag@SeO_2_ bmNPs were determined in response to each run condition. The laboratory-recorded data were statistically analyzed to define the significant factors and the goodness of the BBD model, in this connection, the analysis of variance (ANOVA) and determination coefficient (*R*^2^) were calculated. Furthermore, the association between the four tested variables and Ag@SeO_2_ bmNPs biosynthesis, as well as the prediction of the optimum level of each variable were elucidated. The function of the second-order polynomial quadratic model (eqn (1)) was applied:

1 *Y* = *β*_0_ + ∑*β*_*i*_*X*_*i*_ + ∑*β*_*ij*_*X*_*i*_*X*_*j*_ + *β*_*ii*_*X*_*i*_^2^

where *Y* is the predicted lipase, *β*_0_ is the model constant, *β*_*i*_ is the linear coefficient, *β*_*ij*_ is the cross-product coefficient, *β*_*ii*_ is the quadratic coefficient, and *X*_*i*_, and *X*_*j*_ are the tested variables.

**Table 1:** Box–Behnken and ANN model optimization of Ag@SeO_2_ nanoparticles biosynthesis derived from turmeric extract using a four-factor matrix

Run	Investigated variable^a^				Ag@SeO_2_ bmNP (μg mL^−1^)					
					Actual	Box–Behnken		ANN^b^		
	*X*_1_	*X*_2_	*X*_3_	*X*_4_		Predicted	Error	Predicted	Error	Validation
1	4	40	3	8.047	456.86	417.05	39.81	452.32	4.54	Validation
2	10	40	3	8.047	413.65	402.16	11.49	412.78	0.87	Validation
3	4	80	3	8.047	418.30	400.82	17.48	417.23	1.07	Training
4	10	80	3	8.047	403.44	414.18	−10.74	397.29	6.15	Validation
5	7	60	1	3.714	932.40	866.21	66.19	926.87	5.53	Validation
6	7	60	5	3.714	753.55	663.56	89.99	748.66	4.89	Validation
7	7	60	1	12.380	206.80	267.35	−60.55	206.39	0.41	Validation
8	7	60	5	12.380	173.87	210.97	−37.10	171.23	2.64	Training
9	4	60	3	3.714	872.98	859.65	13.33	873.03	−0.05	Validation
10	10	60	3	3.714	843.30	843.08	0.22	841.98	1.32	Validation
11	4	60	3	12.380	280.59	318.11	−37.52	274.32	6.27	Training
12	10	60	3	12.380	282.50	333.16	−50.66	283.80	−1.30	Training
13	7	40	1	8.047	441.89	389.16	52.73	438.73	3.16	Validation
14	7	80	1	8.047	443.58	384.51	59.07	446.96	−3.38	Validation
15	7	40	5	8.047	160.40	257.09	−96.69	168.75	−8.35	Validation
16	7	80	5	8.047	167.25	257.54	−90.29	172.06	−4.81	Training
17	4	60	1	8.047	573.93	657.41	−83.48	576.78	−2.85	Validation
18	10	60	1	8.047	442.37	480.20	−37.83	444.61	−2.24	Training
19	4	60	5	8.047	397.59	351.45	46.14	397.97	−0.38	Training
20	10	60	5	8.047	601.00	527.13	73.87	612.51	−11.51	Validation
21	7	40	3	3.714	756.90	849.42	−92.52	752.61	4.29	Training
22	7	80	3	3.714	421.35	489.37	−68.02	419.29	2.06	Validation
23	7	40	3	12.380	57.00	−34.26	91.26	55.61	1.39	Training
24	7	80	3	12.380	422.59	321.59	101.00	425.11	−2.52	Training
25	7	60	3	8.047	395.07	414.82	−19.75	415.49	−20.42	Training
26	7	60	3	8.047	388.56	414.82	−26.26	415.49	−26.93	Validation
27	7	60	3	8.047	443.09	414.82	28.27	415.49	27.60	Training
28	4	40	3	8.047	445.85	417.05	28.80	452.32	−6.47	Training
29	10	40	3	8.047	401.62	402.16	−0.54	412.78	−11.16	Validation
30	4	80	3	8.047	406.50	400.82	5.68	417.23	−10.73	Training
31	10	80	3	8.047	397.41	414.18	−16.77	397.29	0.12	Training
32	7	60	1	3.714	915.20	866.21	48.99	926.87	−11.67	Training
33	7	60	5	3.714	747.52	663.56	83.96	748.66	−1.14	Training
34	7	60	1	12.380	198.40	267.35	−68.95	206.39	−7.99	Training
35	7	60	5	12.380	162.81	210.97	−48.16	171.23	−8.42	Training
36	4	60	3	3.714	866.94	859.65	7.29	873.03	−6.09	Validation
37	10	60	3	3.714	835.10	843.08	−7.98	841.98	−6.88	Validation
38	4	60	3	12.380	269.54	318.11	−48.57	274.32	−4.78	Training
39	10	60	3	12.380	275.21	333.16	−57.95	283.80	−8.59	Validation
40	7	40	1	8.047	435.85	389.16	46.69	438.73	−2.88	Validation
41	7	80	1	8.047	433.45	384.51	48.94	446.96	−13.51	Training
42	7	40	5	8.047	145.70	257.09	−111.39	168.75	−23.05	Training
43	7	80	5	8.047	161.54	257.54	−96.00	172.06	−10.52	Training
44	4	60	1	8.047	573.93	657.41	−83.48	576.78	−2.85	Training
45	10	60	1	8.047	435.35	480.20	−44.85	444.61	−9.26	Training
46	4	60	5	8.047	389.45	351.45	38.00	397.97	−8.52	Training
47	10	60	5	8.047	601.00	527.13	73.87	612.51	−11.51	Training
48	7	40	3	3.714	748.60	849.42	−100.82	752.61	−4.01	Validation
49	7	80	3	3.714	421.35	489.37	−68.02	419.29	2.06	Training
50	7	40	3	12.380	57.00	−34.26	91.26	55.61	1.39	Training
51	7	80	3	12.380	414.63	321.59	93.04	425.11	−10.48	Validation
52	7	60	3	8.047	388.32	414.82	−26.50	415.49	−27.17	Training
53	7	60	3	8.047	367.63	414.82	−47.19	415.49	−47.86	Training
54	7	60	3	8.047	438.67	414.82	23.85	415.49	23.18	Training
55	4	40	3	8.047	467.87	417.05	50.82	452.32	15.55	Training
56	10	40	3	8.047	425.68	402.16	23.52	412.78	12.90	Training
57	4	80	3	8.047	430.40	400.82	29.58	417.23	13.17	Training
58	10	80	3	8.047	409.46	414.18	−4.72	397.29	12.17	Validation
59	7	60	1	3.714	949.60	866.21	83.39	926.87	22.73	Training
60	7	60	5	3.714	759.57	663.56	96.01	748.66	10.91	Training
61	7	60	1	12.380	214.11	267.35	−53.24	206.39	7.72	Training
62	7	60	5	12.380	184.89	210.97	−26.08	171.23	13.66	Training
63	4	60	3	3.714	878.96	859.65	19.31	873.03	5.93	Training
64	10	60	3	3.714	851.20	843.08	8.12	841.98	9.22	Training
65	4	60	3	12.380	291.63	318.11	−26.48	274.32	17.31	Validation
66	10	60	3	12.380	289.63	333.16	−43.53	283.80	5.83	Training
67	7	40	1	8.047	447.94	389.16	58.78	438.73	9.21	Validation
68	7	80	1	8.047	453.67	384.51	69.16	446.96	6.71	Training
69	7	40	5	8.047	175.80	257.09	−81.29	168.75	7.05	Training
70	7	80	5	8.047	173.42	257.54	−84.12	172.06	1.36	Training
71	4	60	1	8.047	586.51	657.41	−70.90	576.78	9.73	Training
72	10	60	1	8.047	449.52	480.20	−30.68	444.61	4.91	Training
73	4	60	5	8.047	405.64	351.45	54.19	397.97	7.67	Training
74	10	60	5	8.047	642.23	527.13	115.10	612.51	29.72	Training
75	7	40	3	3.714	764.50	849.42	−84.92	752.61	11.89	Validation
76	7	80	3	3.714	394.87	489.37	−94.50	419.29	−24.42	Training
77	7	40	3	12.380	38.75	−34.26	73.01	55.61	−16.86	Training
78	7	80	3	12.380	430.82	321.59	109.23	425.11	5.71	Training
79	7	60	3	8.047	453.06	414.82	38.24	415.49	37.57	Training
80	7	60	3	8.047	432.64	414.82	17.82	415.49	17.15	Validation
81	7	60	3	8.047	426.33	414.82	11.51	415.49	10.84	Training

a *X*_1_; pH, *X*_2_; bioconversion temperature (°C), *X*_3_; bioconversion time (h), *X*_4_; concentration of turmeric (mg mL^−1^).

b For ANN, the training group was composed of 54, and the validation dataset was composed of 27 runs.

#### 2.4.2. Modeling Ag@SeO_2_ bmNPs biosynthesis using ANN

An important component of the supervised machine learning approach is the historical data. Therefore, the data of the BBD matrix were employed for the machine learning process to produce the prediction model. The dataset was used to train a fully connected ANN. The model architecture employed a layered structure, comprising an input layer, one or more hidden layers, and an output layer. The input nodes directly corresponded to the four experimental parameters under investigation. The output node, singular in nature, represented the biosynthesis of Ag@SeO_2_ bio-nanoparticles. Within the nodes, a hyperbolic tangent sigmoid (NTanH) activation function was implemented for signal processing. The predictive algorithm utilized a fully connected, feedforward multilayer perceptron structure.

Data handling employed a three-pronged approach. The first dataset served as the training ground, where backpropagation optimized network parameters by minimizing error at each neuron. This facilitated robust learning and initial weight establishment. The second dataset fulfilled a validation role, allowing for training interruptions and optimal model selection to prevent overfitting. Finally, a dedicated “outer” dataset held back from training assessed the model's true predictive strength in a final evaluation step, ensuring independent and rigorous performance benchmarks.

The model architecture revolved around a three-layered setup; an input layer, a hidden layer (or multiple layers as determined), and an output layer. Four input neurons mapped directly to the experimental parameters – pH, bioconversion temperature, time, and turmeric concentration. The single output neuron represented the predicted Ag@SeO_2_ bio-nanoparticle biosynthesis. Determining the optimal hidden layer configuration involved an iterative process.

For effective Ag@SeO_2_ bio-nanoparticle biosynthesis modeling, the data were strategically and randomly partitioned into training and validation datasets employing a holdback propagation ratio of 0.3333, indicating that approximately one-third of the data was reserved for validation purposes for rigorous validation. The training dataset comprised 54 runs, which were utilized to minimize prediction error and establish neural weights. Subsequently, a separate validation dataset consisting of 27 runs was employed to monitor the performance of the ANN during training and to determine the optimal stopping point, thereby selecting the finest model. This approach ensured that the ANN did not over fit the training data and generalized well to unseen conditions. Additionally, a third external dataset was reserved for testing the robustness of the ANN model and evaluating its predictive competencies on independent data. To prevent overfitting and improve the generalization of the model, the regularization technique was employed, including the squared penalty method. This method penalizes overly complex models by adding a regularization term to the loss function, encouraging the ANN to prioritize simpler solutions that generalize better to new data.

During training, a learning rate of 0.1 was utilized to facilitate efficient convergence and optimal performance of the ANN model. This learning rate was determined through trial-and-error testing across 5000 training iterations per phase, where various ANN parameters such as the learning rate and hidden layer neuron count were optimized. This meticulous approach yielded the ideal network structure for the best model, ensuring robust performance in predicting Ag@SeO_2_ bio-nanoparticle biosynthesis.

Training continued until a combination of minimal error, including root average squared error and mean absolute deviation, accompanied by maximized *R*-squared values, was achieved. These performance metrics signified the peak performance of Ag@SeO_2_ bio-nanoparticle biosynthesis prediction, with model outputs closely resembling actual experimental values. The network's predictive accuracy was rigorously assessed by comparing its predictions with experimental data and evaluating its ability to generalize to unseen samples, thereby ensuring the reliability and validity of the ANN model.

#### 2.4.3. Experimental verification

The model's predicted optimal settings for maximizing Ag@SeO_2_ bmNPs biosynthesis were translated into triplicate laboratory experiments. Comparing the actual yield with the forecast validated the model's predictive accuracy.

#### 2.4.4. Statistical procedure, and software

The design and statistical analysis of BBD and ANN topology as well as the model construction were performed using the statistical analysis software package; JMP Pro.® (Version 17, SAS Institute Inc., Cary, NC). The alpha value at ≤0.05 was considered the threshold of significance. Training of ANN was performed using 54 runs randomly selected by the software, whereas 27 runs were used to check the validity of the trained ANN model.

### 2.5. Phytochemical analyses

#### 2.5.1. Folin–Ciocalteu assay

Folin–Ciocalteu (FC) reagent was prepared by dilution in distilled water (1 : 9) according to the manufacturer's instructions. The FC reagent (5 mL) was added to each tube containing the sample (100 μL) and mixed gently. After 3 minutes, sodium carbonate solution (4 mL, 75 g L^−1^) was added and the mixture was well-mixed. The final volume was adjusted by distilled water to 10 mL. The tubes were incubated in the dark at 40 °C for 30 minutes. This allows the phenolics in the samples to react with the FC reagent and develop a blue color. A blank was prepared using the same previous steps but without any sample material. A standard curve was prepared from a series of standard (reference) solutions using gallic acid with concentrations ranging from 0 to 200 μg mL^−1^. After incubation, the absorbance of each tube was measured at a wavelength of 765 nm.^45^ The gallic acid standard curve (*y* = 0.0062*x*, *r*^2^ = 0.987) was applied to calculate the phenolic contents as milligrams of gallic acid equivalents per gram of sample (mg GAE per g).

#### 2.5.2. Aluminum chloride assay

100 μL of each tested sample was added to a test tube containing distilled water (4 mL). Sodium nitrite solution (0.3 mL, 5%, 0.05 g mL^−1^) was added to the mixture. After 5 minutes, freshly prepared aluminum chloride ethanol solution (0.3 mL, 10%, 0.1 g mL^−1^) was added to each tube. After 1 minute, sodium hydroxide solution (1 M, 2 mL) was added. The final volume was adjusted by distilled water to 10 mL. A blank was prepared using the same previous steps but without any sample material. A standard curve was prepared from a series of standard (reference) solutions using catechin acid with concentrations ranging from 0 to 200 μg mL^−1^. The absorbance of each tube was measured at a wavelength of 510 nm.^46^ The flavonoid values were calculated as milligrams of catechin equivalents per gram of sample (mg CE per g) using a catechin standard curve (*y* = 0.0028*x*, *r*^2^ = 0.988).

### 2.6. Potential antioxidant activity

#### 2.6.1. ABTS assay

To perform the ABTS assay^47^ to measure antioxidant activity, the following steps are typically followed: prepare the ABTS radical solution by reacting ABTS with potassium persulfate. The ABTS radical solution is then incubated for 16–24 hours at room temperature in the dark to allow the radical to form. A serial dilution of each sample was prepared by dilution in methanol. ABTS solution was added to each sample tube and well-mixed then incubate the mixture for 30 minutes at room temperature in the dark. The samples' absorbance was measured at 734 nm and the % inhibition was calculated from the next eqn (2):

2 Inhibition (%) = [(Abs.(control) − Abs.(sample))/Abs.(control)] × 100

The antioxidant activity can also be expressed as an IC_50_ value, which is the concentration of the antioxidant sample required to inhibit the ABTS radical by 50%.

#### 2.6.2. DPPH assay

DPPH solution (1 mM) was prepared by dissolving 40 mg of DPPH in 100 mL of methanol. Ascorbic acid solution (0.1 mM) was prepared by dissolving 10 mg of ascorbic acid in 100 mL of distilled water. The DPPH stock solution (1 mM) was diluted with methanol to obtain a working solution with an absorbance of approximately 1.0 at 517 nm. In a clean test tube, add the following: 3 mL of DPPH working solution, and 100 μL of sample solution. The mixture was vortexed thoroughly and incubated in the dark for 30 minutes at room temperature. The absorbance of the mixture was measured at 517 nm using a spectrophotometer. The percentage of DPPH scavenging activity was calculated using the following formula (eqn (3)):^48^

3 DPPH scavenging activity (%) = [(Abs.(control) − Abs.(sample))/Abs.(control)] × 100

where: Abs.(control) is the absorbance of the DPPH working solution without a sample. Abs.(sample) is the absorbance of the reaction mixture.

### 2.7. Antibacterial activity

#### 2.7.1. Bacteria

Four species of bacterial Gram-positive (*Staphylococcus aureus* (ATCC 6538) & *Bacillus cereus* (EMCC number 1080)) and Gram-negative (*Escherichia coli* (ATCC 10536) & *Klebsiella pneumoniae* (ATCC 10031)) strains were assessed.

#### 2.7.2. Agar well-diffusion assay

DMSO was used as a negative control and Cefotaxime was used as a positive control. To assess the antibacterial activity of the turmeric extract and Ag@SeO_2_ NPs, a sterile Mueller–Hinton agar plate was inoculated with the target bacteria using a sterile swab. A sterile cork borer was used to create a 6 mm diameter well in the center of the agar plate. Approximately 100 μL of each sample was carefully pipetted into the well. The inoculated agar plates were then incubated at 37 °C for 24 hours. After incubation, the diameter of the clear zone surrounding the well, indicating bacterial inhibition, was measured.^49^

#### 2.7.3. The minimum inhibitory concentration (MIC)

To determine the MIC of the sample, serial dilutions were prepared in nutrient broth, encompassing a concentration range of 21 200 to 82.8125 μg mL^−1^. The control consisted of solely inoculated broth and was incubated alongside the dilutions for 24 hours at 37 °C. The absence of visible growth in a tube defined the MIC endpoint. This finding was validated by visually comparing turbidity before and after incubation and further supported by measuring the OD at *λ* = 600 nm.^50^

## 3. Results and discussion

### 3.1. Characterization of the nanocomposite

#### 3.1.1. FT-IR spectral analyses

**Fig. 1:**
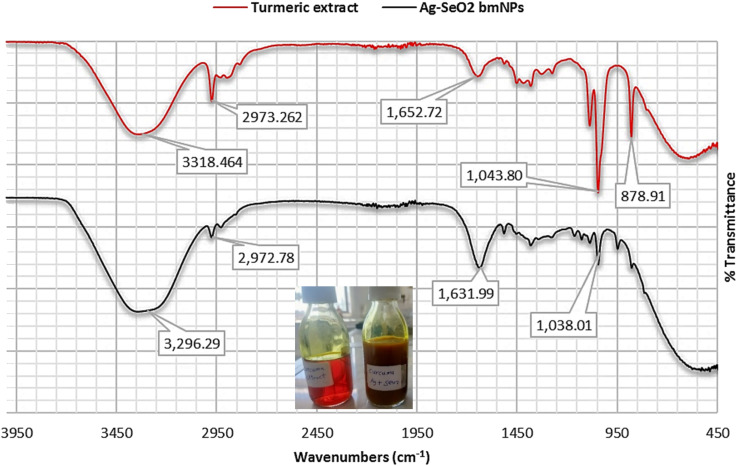
The FT-IR spectral analyses of turmeric extract and Ag@SeO_2_ bmNPs.

The FT-IR spectral data of the turmeric extract and the Ag@SeO_2_ bmNPs nanocomposite are shown in Fig. 1 and Table S1.† In the analysis of turmeric extract, the strong absorption band at 3318 cm^−1^ reveals abundant hydroxyl (OH) groups in phenolics and alcohols, suggesting potential antioxidant activity in the turmeric extract. Stretching vibration at 2973 cm^−1^ indicates the presence of aliphatic groups, the essential building blocks of many organic molecules within the extract. The absorption band at 2162 cm^−1^ hints at the presence of unsaturated compounds, which might contribute to the vibrant color and diverse bioactivities of turmeric. The absorption band at 1652 cm^−1^ suggests the presence of conjugated ketones or aromatic carboxylic acids, potentially offering medicinal properties. Bending vibrations of C–H bonds in aromatic groups at 1381 cm^−1^ confirm the aromatic nature of some components in the extract. The absorption bands at 1087 and 1043 cm^−1^ reveal the presence of carbohydrates and polysaccharides, indicating a diverse range of biomolecules beyond just aromatic compounds. In-plane bending of C–H bonds within aromatic rings is indicated by the 878 cm^−1^ band, further confirming the aromatic nature of some components. The absorption band at 592 cm^−1^ signifies the presence of aromatic skeletal vibrations, solidifying the presence of aromatic compounds within the extract. The 430 cm^−1^ absorption band remains a bit of a mystery, potentially arising from out-of-plane bending of hydroxyl groups or stretching vibrations of aromatic C–C and C–N bonds.

On the other hand, the FT-IR spectrum of Ag@SeO_2_ bmNPs revealed that broader O–H stretching vibrations at 3338 and 3296 cm^−1^, compared to the extract, suggest interactions between the hydroxyl groups and the surface functional groups of the nanoparticles. The presence of aliphatic groups in the nanoparticles is confirmed by the similar absorption band at 2972 cm^−1^, indicating they remain after bio-synthesis. A slight shift in the 2148 cm^−1^ absorption band further supports the notion of interactions between the biomolecules and the nanoparticles' surface. The shifted C=O stretching absorption band at 1631 cm^−1^ hints at potential coordination between these groups and metal ions on the nanoparticles' surface. A new absorption band at 1515 cm^−1^ suggests adsorption on the nanoparticles, indicating they interact with various biomolecules. Just like in the extract, the 1381 cm^−1^ absorption band confirms the presence of aromatic CH groups in the nanoparticles. Similar to the extract, the absorption bands at 1038 and 1045 cm^−1^ suggest that carbohydrates/polysaccharides are still present in the nanoparticles. The exciting new absorption band at 947 cm^−1^ potentially arises from Se–O stretching vibrations, confirming the presence of SeO_2_ in the bio-synthesized nanoparticles. Stretching vibrations between metal (Se) and oxygen atoms might be responsible for the absorption band at 486 cm^−1^, further supporting the presence of the desired nanoparticles.

By analyzing these absorption bands, we gain valuable insights into the molecular structure of both the turmeric extract and the bio-synthesized Ag@SeO_2_ nanoparticles. The FT-IR spectra confirm the presence of functional groups associated with biomolecules like phenolics, carbohydrates, and proteins in both the turmeric extract and the Ag@SeO_2_ bmNPs. The broader and shifted absorption bands in the bmNPs spectrum compared to the extract suggest interactions between the biomolecules and the nanoparticles' surface.^51^ These interactions could involve hydrogen bonding, electrostatic interactions, or coordination with metal ions, potentially contributing to the bio-reduction and stabilization of the nanoparticles.^52^ The presence of new absorption bands in the bmNPs spectrum, like the amide band and the Se–O stretching vibration, further supports the hypothesis of biomolecule adsorption and SeO_2_ formation on the nanoparticle surface.

#### 3.1.2. Zeta potential and zeta-sizer analyses of Ag–SeO_2_ bmNPs

**Fig. 2:**
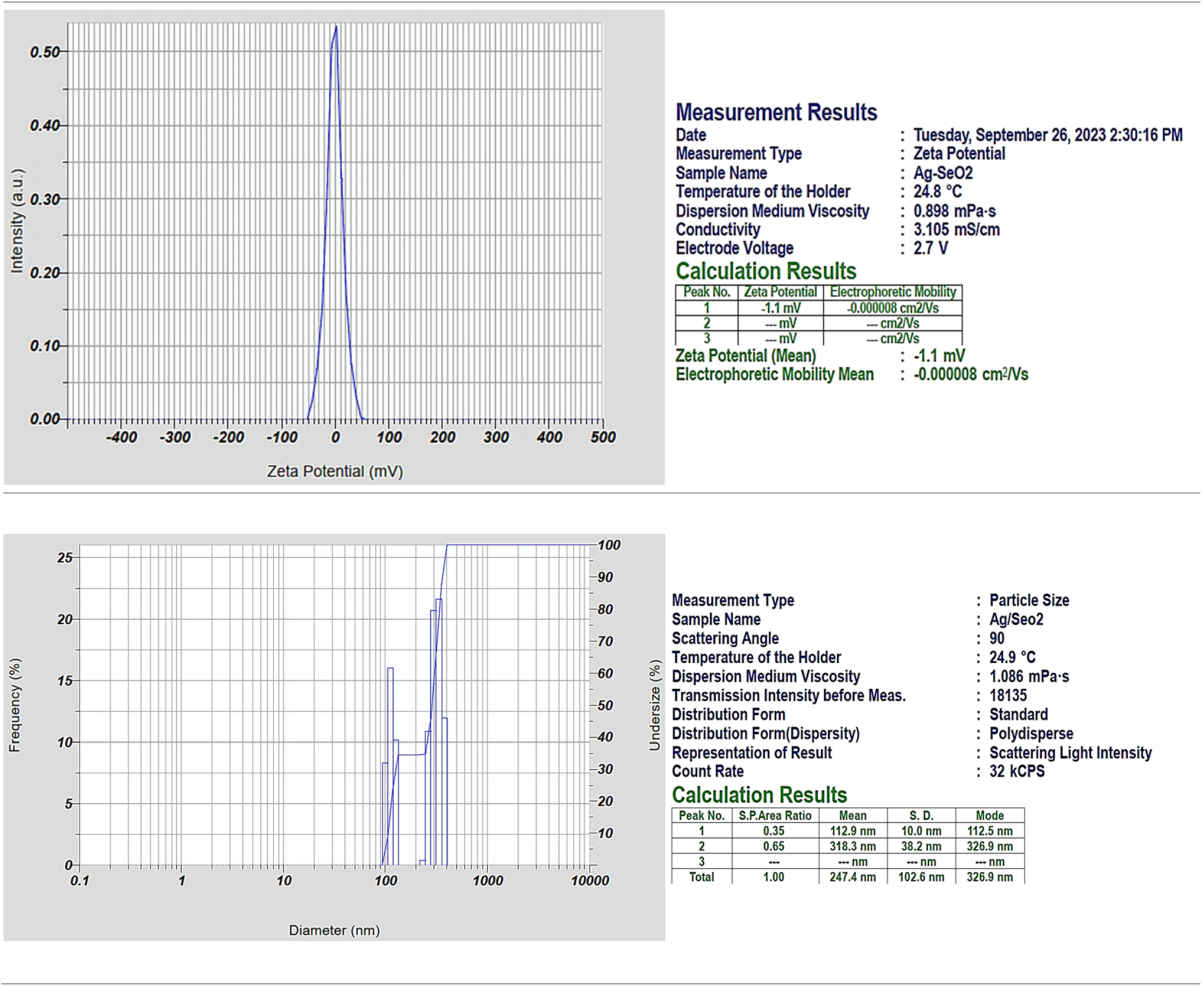
The chart of zeta potential and zeta sizer analyses of Ag@SeO_2_ bmNPs.

The zeta potential of −1.1 mV for the Ag@SeO_2_ bmNPs prepared from turmeric ethanol extract indicates that the nanoparticles have a slightly negative surface charge (Fig. 2). This negative surface charge is likely due to the presence of functional groups on the surface of the nanoparticles, such as hydroxyl groups. The negative surface charge can help to prevent the nanoparticles from aggregating and can also help to improve their stability in solution. The mean electrophoretic mobility of −0.000008 cm^2^ V^−1^ s^−1^ is also consistent with the negative surface charge of the nanoparticles. Electrophoretic mobility is the rate at which charged particles move in an electric field.

The negative electrophoretic mobility indicates that the nanoparticles are moving in the opposite direction of the electric field, which is consistent with the fact that they have a negative surface charge (Fig. 2). Particularly, the zeta potential results suggest that the Ag@SeO_2_ bmNPs have a slightly negative surface charge and are stable in solution. Here are some additional thoughts on the zeta potential result: the negative zeta potential of the Ag@SeO_2_ bmNPs is likely due to the presence of functional groups on the surface of the nanoparticles, such as hydroxyl groups.^53^ These functional groups can dissociate in water, resulting in a negatively charged surface. The negative zeta potential of the Ag@SeO_2_ bmNPs can help to prevent the nanoparticles from aggregating.^54^ This is because nanoparticles with the same charge repel each other. The negative zeta potential can also help to improve their stability in solution. This is because the negative charge on the surface of the nanoparticles attracts water molecules, which creates a hydration layer around the nanoparticles. This hydration layer helps to protect the nanoparticles from aggregation and precipitation.

Dynamic light scattering (DLS) analysis revealed a polydisperse size distribution for Ag@SeO_2_ nanoparticles, indicating particles of various sizes. Two distinct peaks in the size distribution were observed: peak 1: smaller particles with an average size of 112.9 nm (SD 10 nm), likely representing individual Ag nanoparticles or small Ag@SeO_2_ composites. Peak 2: larger particles with an average size of 318.3 nm (SD 38.2 nm), likely representing larger Ag@SeO_2_ composite nanoparticles or even aggregates. The presence of two distinct populations and a broad size distribution (indicated by a PI value of 1.243) is further supported by the size distribution plot diameter. Analyzing the size distribution can help us optimize the synthesis process to achieve desired nanoparticle sizes for specific applications. By studying the factors influencing the distribution (*e.g*., precursor concentrations, reaction time, temperature), we can tailor the synthesis to obtain nanoparticles with optimal antibacterial or antioxidant properties.

#### 3.1.3. TEM

**Fig. 3:**
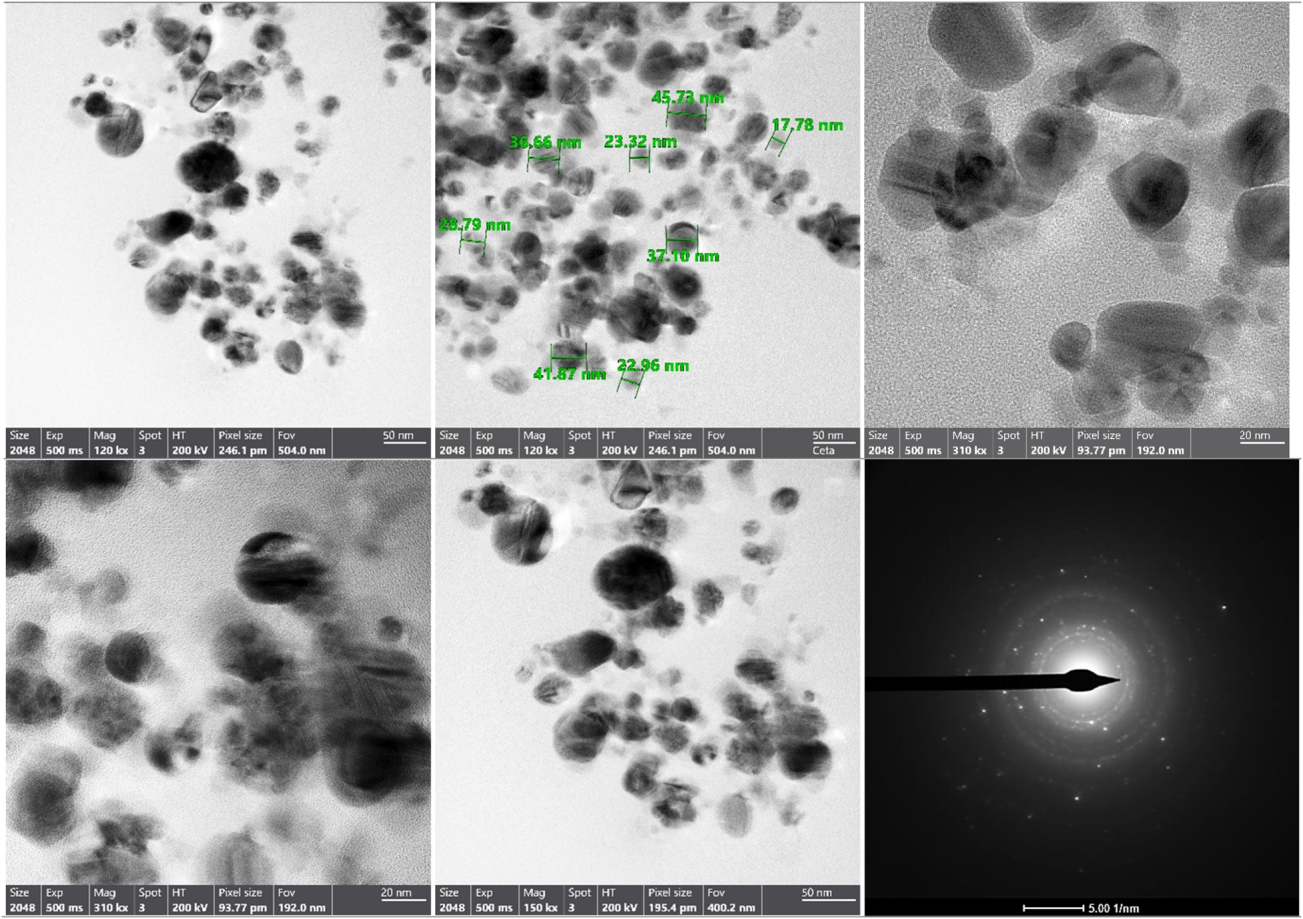
The TEM image, and selected area diffraction pattern of Ag@SeO_2_ bmNPs.

The results of TEM (Fig. 3) indicate that the bimetallic nanoparticles solution (Ag@SeO_2_ bmNPs) prepared from turmeric ethanol extract is composed of spherical nanoparticles with an average particle size of approximately 20 nm (Table S2†). The most common particle size range is 10–20 nm, followed by 21–30 nm and 31–40 nm. There are a few particles in the 41–50 nm and 51–60 nm ranges, and one particle larger than 60 nm. The selected area diffraction pattern shows bright spots, which indicates that the nanoparticles are crystalline.

The percent polydispersity of the Ag@SeO_2_ bmNPs is approximately 25%. This is relatively high, indicating that the Ag@SeO_2_ bmNPs are polydisperse. The interparticle forces between nanoparticles are dominated by van der Waals forces.^55^ These forces are attractive and can lead to the aggregation of the bmNPs. The surface chemistry of Ag@SeO_2_ bmNPs can also affect their aggregation. Hydrophilic bmNPs are less likely to aggregate than hydrophobic bmNPs. At higher concentrations, Ag@SeO_2_ bmNPs are more likely to collide and aggregate. In addition, a higher temperature can increase the kinetic energy of the nanoparticles, which can lead to an increased aggregation rate.^56^ The nanoparticles that are too small may be difficult to synthesize and characterize, while nanoparticles that are too large may not be able to penetrate cells or tissues. Crystalline nanoparticles have a well-defined structure, which gives them specific properties that can be exploited for different applications. For instance, crystalline nanoparticles may be more stable and durable than non-crystalline nanoparticles.

#### 3.1.4. XRD

**Fig. 4:**
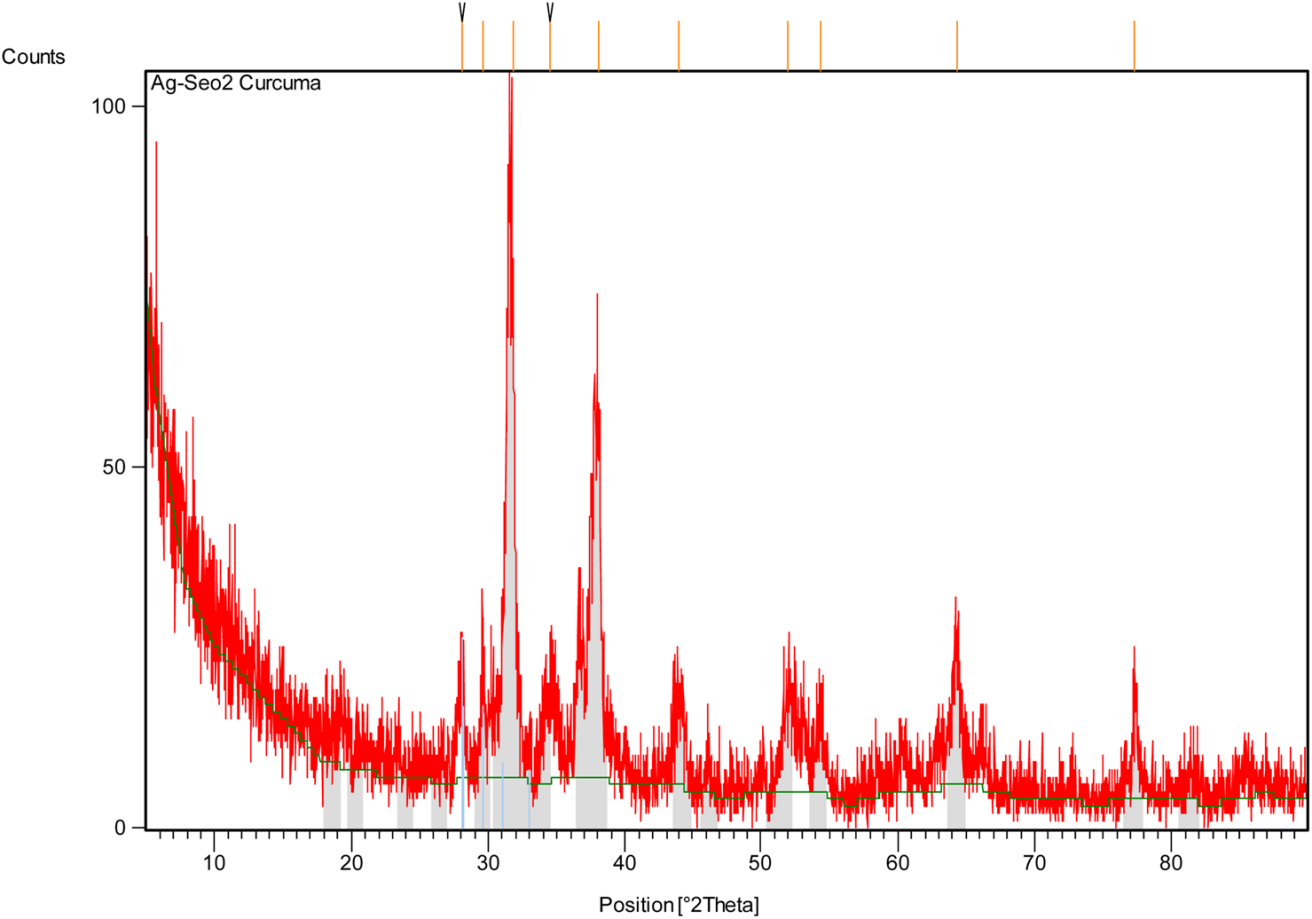
The XRD pattern of Ag@SeO_2_ bmNPs.

The XRD analysis of Ag@SeO_2_ bmNPs (Table S3 and Fig. 4, S1†) shows that the bmNPs are crystalline. The presence of sharp peaks in the XRD pattern indicates that the nanoparticles have a well-defined crystal structure.^57^ The *d*-spacing values of the peaks correspond to the following crystal planes of Ag and SeO_2_: Ag: (111), (200), and (220), and SeO_2_: (112) and (211). The relative intensity of the peaks suggests that the Ag@SeO_2_ bmNPs are composed of a single layer of Ag NPs on a SeO_2_ core. This core–shell structure is consistent with the EDX analysis. The XRD analysis also provides information about the average size of the Ag@SeO_2_ bmNPs. The average crystallite size can be calculated using the Scherrer eqn (4):

4 *D* = *K* × *λ*/(*β* × cos(*θ*))

where: *D* is the average crystallite size in nm, *K* is the Scherrer constant (typically 0.9), *λ* is the wavelength of the X-ray radiation (typically 1.5418 Å for Cu Kα radiation), *β* is the full width at half maximum (FWHM) of the peak in radians, and *θ* is the Bragg angle in degrees.

Using the Scherrer eqn (4), the average crystallite size of the Ag@SeO_2_ bmNPs was calculated to be approximately 20 nm. This is in good agreement with the size of the Ag@SeO_2_ bmNPs observed in TEM images. The FWHM values are generally small, indicating that the peaks are well-defined^58^ and that the Ag@SeO_2_ bmNPs have a high degree of crystallinity. The FWHM values increase slightly with decreasing *d*-spacing. This is expected, as smaller crystals tend to have broader peaks. The *d*-spacing values correspond to the crystal planes of Ag and SeO_2_, confirming the presence of both materials in the Ag@SeO_2_ bmNPs. The relative intensity values indicate that the (111) and (200) crystal planes of Ag are the most dominant in the Ag@SeO_2_ bmNPs. The relative intensity values of the Ag peaks are generally higher than those of the SeO_2_ peaks. This suggests that the Ag@SeO_2_ bmNPs have a higher surface area of Ag than SeO_2_. The presence of multiple peaks in the XRD pattern indicates that the nanoparticles are polycrystalline, meaning that they are composed of many small crystals. The data suggests that the nanoparticles are well-crystalline and have a core–shell structure with an Ag core and a SeO_2_ shell. The Ag@SeO_2_ bmNPs also have a high surface area of Ag. Mainly, the XRD analysis confirms the crystallinity, core–shell structure, and average size of the Ag@SeO_2_ bmNPs. This information can be used to optimize the biosynthesis process and to develop new applications for Ag@SeO_2_ bmNPs.

Table 2 summarizes the particles' size of Ag@SeO_2_ bmNPs calculated from XRD data using the Scherrer eqn (4). Peaks with similar *d*-spacings seem to have closer particle size estimations. For example, peaks 1 and 3 (both around 2.8 Å) have similar sizes (both around 25.4 nm), while peaks 5, 6, and 8 (around 2.0–2.3 Å) also have comparable sizes (around 14.6 nm).

**Table 2:** The estimated particle size of Ag@SeO_2_ bmNPs calculated from XRD data using the Scherrer equation

Peak number	Peak position (°2*θ*)	FWHM (°2*θ*)	Miller indices	*d*-Spacing (Å)	Calculated particle size (nm)
1	28.0653	0.3149	(111)	3.17945	25.415
2	29.6122	0.2362	(021)	3.01679	30.568
3	31.8716	0.3149	(121)	2.80790	25.415
4	34.4904	0.9446	(031)	2.60046	8.554
5	38.0638	0.6298	(131)	2.36415	14.562
6	44.0496	0.6298	(221)	2.05579	14.562
7	51.9532	0.7872	(230)	1.76011	11.505
8	54.3324	0.6298	(132)	1.68853	14.562
9	64.3039	0.3936	(321)	1.44868	21.569
10	77.3277	0.3840	(400)	1.23297	22.143

#### 3.1.5. EDX

**Fig. 5:**
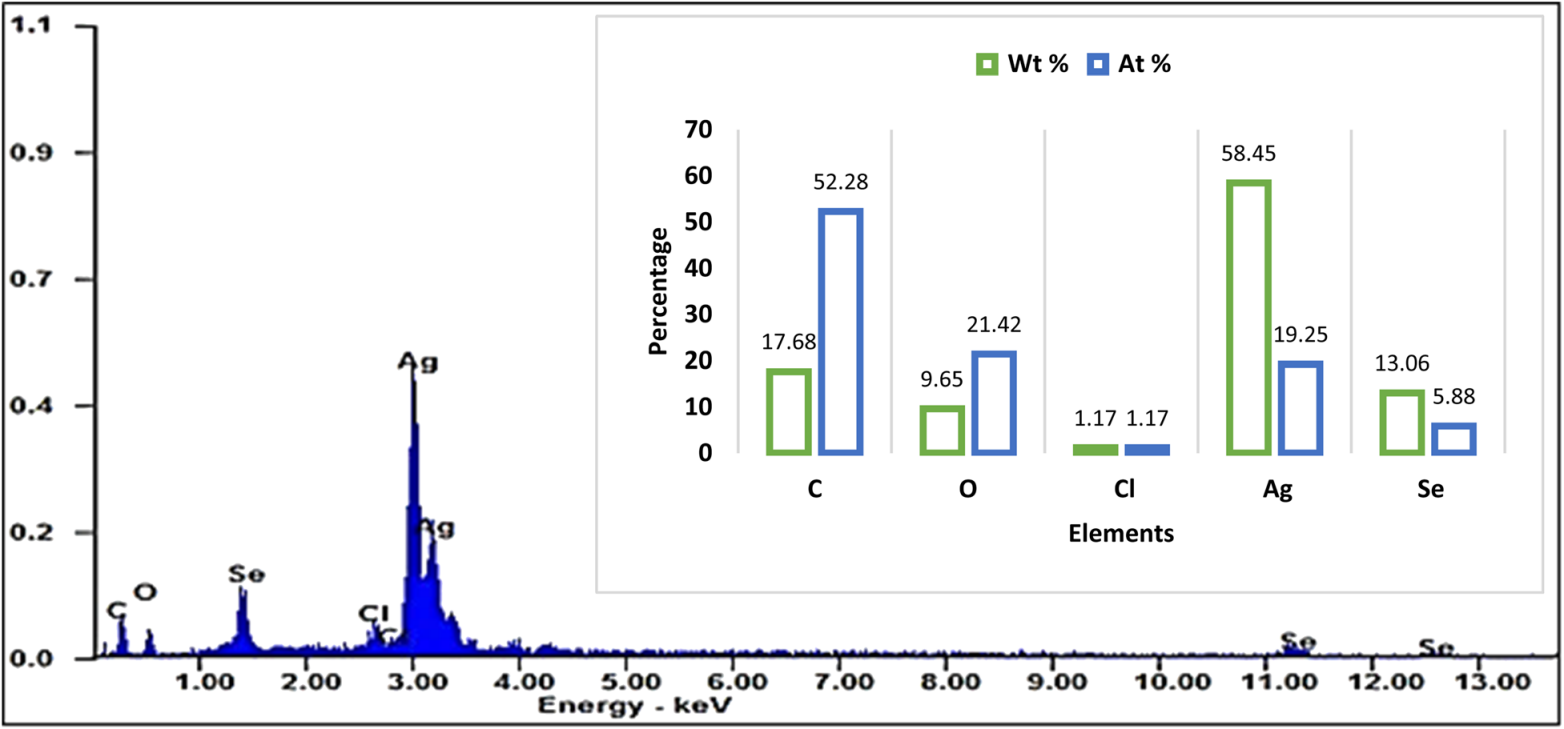
The EDX pattern of Ag@SeO_2_ bmNPs.

The EDX analysis of Ag@SeO_2_ bmNPs shows that the major elements present are carbon (C), oxygen (O), silver (Ag), and selenium (Se). The weight percent (wt%) and atomic percent (at%) of each element are shown in Fig. 5. The high carbon and oxygen content is likely due to the presence of organic matter from the turmeric extract used in the biosynthesis process. The high silver and selenium content confirms the presence of Ag@SeO_2_ bmNPs in the sample. The atomic ratio of Ag to Se is approximately 3 : 1, which is consistent with the stoichiometry of Ag@SeO_2_ bmNPs. Particularly, the occurrence of carbon and oxygen suggests that the Ag@SeO_2_ bmNPs are coated with a layer of organic matter. This organic layer may help to stabilize the Ag@SeO_2_ bmNPs and prevent them from agglomerating.^59^ The high atomic ratio of Ag to Se indicates that the Ag@SeO_2_ bmNPs are composed of a single layer of Ag NPs on a SeO_2_ core. This core–shell structure is expected to give the Ag@SeO_2_ bmNPs unique properties, such as enhanced catalytic activity and antimicrobial activity.

#### 3.1.6. SEM

**Fig. 6:**
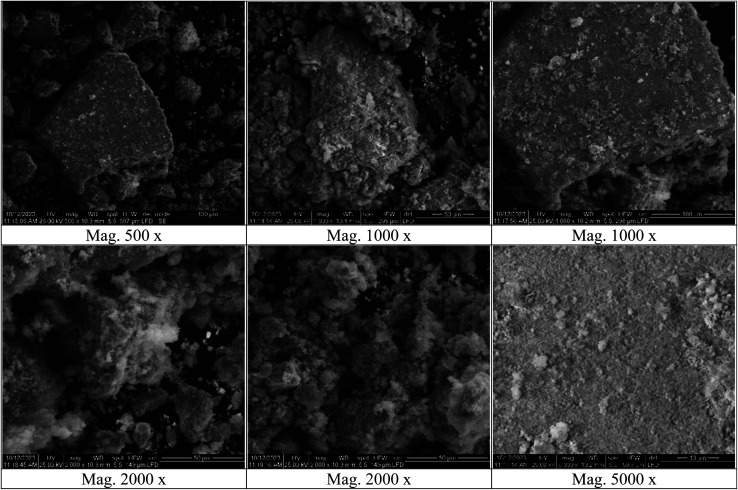
The SEM micrographs with different magnifications of Ag@SeO_2_ bmNPs.

The SEM pattern of the Ag@SeO_2_ bmNPs (Fig. 6) showed that the nanoparticles are spherical and have a smooth surface. The smooth surface of the Ag@SeO_2_ bmNPs suggests that they are well-crystalline. The uniform size of the bimetallic nanoparticles suggests that the biosynthesis process is well-controlled, with an average diameter of approximately 20 nm. The nanoparticles are aggregated to some extent, but the aggregation is not severe. The SEM pattern also shows that the Ag@SeO_2_ nanoparticles are coated with a thin layer of organic material. This organic layer is likely the turmeric extract used in the biosynthesis process. The organic layer may help to stabilize the Ag@SeO_2_ bmNPs and prevent them from aggregating.

Silver nanoparticles might be adsorbed onto the surface of SeO_2_ nanoparticles. The nanoparticles could be individual entities that have clumped together, appearing smooth on the surface. One element might be concentrated on the outer surface, even if not in a perfect shell-like structure. The selenium atoms of the Ag@SeO_2_ bmNPs are likely arranged in a hexagonal closed-packed (hcp) crystal structure.^60,61^ This is the most stable crystal structure for selenium. Furthermore, the Ag@SeO_2_ bmNPs are likely polycrystalline, meaning that they are composed of many small crystals. The size of the crystals is likely in the range of 10–20 nm.

### 3.2. The mechanism of the formation of Ag@SeO_2_ bmNPs

The formation of Ag@SeO_2_ bmNPs core–shell nanoparticles involves a multi-step process that encompasses both reduction and oxidation reactions.^62,63^ The first step is the reduction of AgNO_3_ to Ag-NPs by turmeric extract. Turmeric extract, rich in polyphenolic compounds like curcumin, acts as a natural reducing agent capable of converting silver ions (Ag^+^) to silver nanoparticles (Ag-NPs) in silver nitrate (AgNO_3_) solution. The hydroxyl groups and methoxy groups present in curcumin can donate electrons to Ag^+^, leading to the formation of Ag atoms and the stabilization of Ag-NPs by curcumin molecules. This reduction reaction can be represented as:2AgNO_3_ + C_14_H_16_O_6_(curcumin) → 2Ag + 2HNO_3_ + C_14_H_16_O_6_(oxidized curcumin)

The second step is the formation of Ag@SeO_2_ bmNPs. In which, selenium dioxide (SeO_2_) dissociates in water to form selenite ions (SeO_3_^2−^). Ag-NPs (formed in the first step) interact with SeO_3_^2−^ where Ag-NPs acts as a reducing agent towards SeO_3_^2−^ ions. The SeO_3_^2−^ ions are reduced to selenide ions (Se^2−^) and adsorbed onto the surface of the Ag-NPs. This reduction reaction can be represented as:Ag + SeO_3_^2−^ → Ag@SeO_2_ bmNP + Se^2−^

During the formation of Ag@SeO_2_ bmNP, the adsorbed Se^2−^ ions undergo oxidation in the presence of oxygen (O_2_) to form a thin layer of SeO_2_ on the surface of the Ag-NPs, resulting in the formation of Ag@SeO_2_ bmNPs core–shell nanoparticles. This oxidation reaction can be represented as:Se^2−^ + O_2_ → SeO_2_

The overall process can be summarized by the following simplified reactions:2AgNO_3_ + C_14_H_16_O_6_(curcumin) → 2Ag + 2HNO_3_ + C_14_H_16_O_6_(oxidized curcumin)Ag + SeO_3_^2−^ → Ag@SeO_2_ bmNP + Se^2−^Se^2−^ + O_2_ → SeO_2_

Factors influencing the formation and properties of Ag@SeO_2_ bmNPs core–shell nanoparticles include the concentration of turmeric extract, AgNO_3,_ and SeO_2_; temperature and reaction time; pH of the solution, and the presence of stabilizing or capping agents (*e.g.*, turmeric extract). The resulting Ag@SeO_2_ bmNPs core–shell nanoparticles exhibit unique properties due to the synergistic combination of AgNPs and SeO_2_, making them promising materials for various applications. The next investigation was to optimize the biosynthesis process of Ag@SeO_2_ bmNPs.

### 3.3. Modeling Ag@SeO_2_ bmNPs biosynthesis by BBD

The responded data in Table 1 present the results of a BBD experiment for the Ag@SeO_2_ bmNPs synthesis using turmeric extract. The data demonstrate the influence of four critical factors *i.e.*, pH (*X*_1_), bioconversion temperature (*X*_2_), bioconversion time (*X*_3_), and turmeric concentration (*X*_4_) on the Ag@SeO_2_ bmNPs yield. The relative agreement between the predicted and actual values of BBD validates the model's effectiveness.

**Fig. 7:**
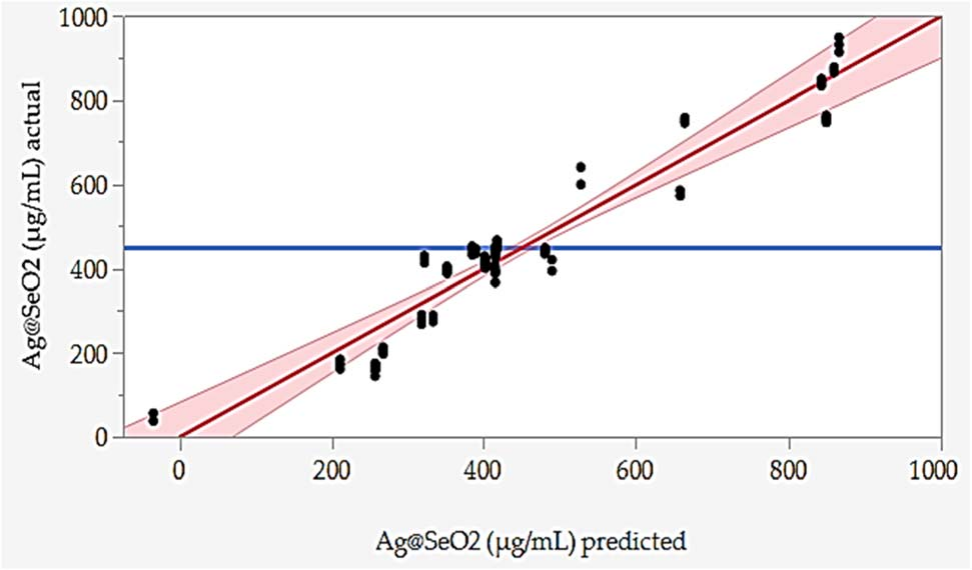
Predicted *versus* actual values of Ag@SeO_2_ bmNPs biosynthesis by turmeric extract.

Furthermore, the error analysis shows the difference between the actual and predicted values, indicating how well the model fits the data. The model predictions generally agree with the actual values in most points, as evidenced by the overall small error values. However, some runs show obvious discrepancies between predicted and actual values, such as runs 24, 42, 43, 48, 72, and 78. This suggests that the model may need further refinement. However, the linear relationship between the predicted and actual points was plotted (Fig. 7).

The BBD model displayed points that aligned with the prediction line. Nevertheless, linear regression demonstrated that several points are notably located away from the prediction line. Overall, the data suggests an obvious relationship between the factors and Ag@SeO_2_ bmNPs production, as evidenced by the variability in the actual values. These findings suggest the potential of turmeric extract as a natural and efficient reducing agent for Ag@SeO_2_ bmNP synthesis, paving the way for further research into the underlying mechanisms and applications of these biogenic nanoparticles (Fig. 7).

### 3.4. Evaluation of the BBD model

The ANOVA data (Table 3) provides valuable insights into the main and interactive effects of various factors on Ag@SeO_2_ bmNPs biosynthesis using turmeric extract. ANOVA confirms the effectiveness of the BBD in capturing the significant factors influencing Ag@SeO_2_ bmNP biosynthesis using turmeric extract. The model exhibits a good adjusted-*R*^2^ value of 0.9075, indicating that it accounts for 90.75% of the data's variability. Additionally, the predicted-*R*^2^ value of 0.8829 suggests that the model may have a good predictive ability.

**Table 3:** ANOVA for the effect of experimental variables on Ag@SeO_2_ bmNP biosynthesis^a^

Source	Freedom degree	Sum of squares	Mean square	*F*-Ratio	*p-*Value
Model	14	3 682 206	263 015	57.07	<0.001^b^
Linear	4	2 638 530	659 633	143.13	<0.001^b^
*X*_1_	1	5	5	0.00	0.973
*X*_2_	1	40	40	0.01	0.926
*X*_3_	1	150 967	150 967	32.76	<0.001^b^
*X*_4_	1	2 487 518	2 487 518	539.76	<0.001^b^
Square	4	548 469	137 117	29.75	<0.001^b^
*X*_1_ × *X*_1_	1	123 483	123 483	26.79	<0.001^b^
*X*_2_ × *X*_2_	1	141 732	141 732	30.75	<0.001^b^
*X*_3_ × *X*_3_	1	30	30	0.01	0.936
*X*_4_ × *X*_4_	1	117 870	117 870	25.58	<0.001^b^
2-Way interaction	6	495 207	82 534	17.91	<0.001^b^
*X*_1_ × *X*_2_	1	598	598	0.13	0.720
*X*_1_ × *X*_3_	1	93 400	93 400	20.27	<0.001^b^
*X*_1_ × *X*_4_	1	750	750	0.16	0.688
*X*_2_ × *X*_3_	1	19	19	0.00	0.948
*X*_2_ × *X*_4_	1	384 392	384 392	83.41	<0.001^b^
*X*_3_ × *X*_4_	1	16 047	16 047	3.48	0.066
Error	66	304 164	4609		
Lack-of-fit	10	291 185	29 118	125.63	<0.001^b^
Pure error	56	12 979	232		
Total	80	3 986 370			

**Model evaluation statistics**					
Determination coefficient (*R*^2^)			0.9237		
Adjusted-*R*^2^			0.9075		
Predicted-*R*^2^			0.8829		

a *X*_1_; pH, *X*_2_; bioconversion temperature (°C), *X*_3_; bioconversion time (h), *X*_4_; concentration of turmeric extract (mg mL^−1^).

b Significant value.

One of the important model goodness relies on high positive *R*^2^ and adjusted-*R*^2^ values that are ≥0.75 and relatively close.^64,65^ The current model boasts high values (*R*^2^ = 0.9237, adjusted-*R*^2^ = 0.9075), suggesting strong agreement between predicted and experimental results. Increasing *R*^2^ and adjusted-*R*^2^ values generally signify an improved model fit and more accurate relationships between tested variables and the response. However, a high *R*^2^ alone isn't a guarantee of model quality. Only when accompanied by a similarly high adjusted-*R*^2^ and an insignificant lack-of-fit can truly infer a robust regression model.^66^

Regarding the tested variables, two (*X*_3_, and *X*_4_) of the four investigated factors exhibit statistically significant individual effects on Ag@SeO_2_ bmNP production. Notably, turmeric concentration has the most significant impact, with the highest *F* value and a low *p*-value of less than 0.001, highlighting its crucial role in the biosynthesis process. The two-way interaction between bioconversion temperature and turmeric concentration stands out as statistically significant (*p*-value < 0.001), indicating a synergistic effect between these two factors. Similarly, the interaction between pH and bioconversion time shows a significant influence (*p*-value < 0.001). The square effect of bioconversion time was the only insignificant factor.

To decipher the influence of individual variables and their interactions on Ag@SeO_2_ bmNP biosynthesis, statistically significant coefficients are key. Typically, an alpha level of ≤0.05 is set to assess the significance of model terms.^67^ At this threshold, a significant coefficient indicates a less than 5% chance of wrongly concluding a link between a variable and the response when none exists.^66,68^ Unfortunately, our model exhibits a significant lack-of-fit (*p*-value < 0.001), hinting at unaccounted-for factors or potential experimental errors. This highlights the need for further optimization and exploration of additional variables to refine the model's accuracy and predictive power.

The ANOVA results for the quadratic model (Table 3), investigated the influence of experimental variables on Ag@SeO_2_ bmNP biosynthesis. The highly significant model (*p* < 0.001) and high *R*^2^ values (*R*^2^ = 0.9237, adjusted-*R*^2^ = 0.9075) indicate a strong correlation between the variables and the response. However, the significant lack-of-fit (*p* < 0.001) necessitates potential model improvement.

Despite the statistically significant findings of the BBD in capturing the main and interactive effects of factors influencing Ag@SeO_2_ bmNP biosynthesis, the significant lack-of-fit test suggests limits its ability to fully model the complex relationships involved. The lack-of-fit test reveals a significant discrepancy between the predicted values generated by the Box–Behnken model and the actually observed Ag@SeO_2_ bmNP yield (*p*-value < 0.001). This suggests that the model, while informative, doesn't perfectly capture the complex relationships between the investigated factors and the response variable (Ag@SeO_2_ bmNP yield). This necessitates the exploration of alternative modeling approaches to improve the accuracy and predictive capabilities of the model. While the current model provides valuable information about the main and interactive effects of the investigated factors, the lack-of-fit highlights its limitations. So, another modeling approach is necessary to enhance the predictive capabilities and reliability.

### 3.5. Modeling Ag@SeO_2_ bmNP by ANN

Maximizing Ag@SeO_2_ bmNP bioproduction demanded a cutting-edge AI-powered modeling approach. Building upon Box–Behnken design data, a fully connected, multilayer feed-forward ANN was constructed as the predictive core of the neural network platform. Extensive trial-and-error optimization, involving numerous iterations of 5000 training epochs each, yielded the ideal ANN configuration. This optimal model, featuring a squared learning rate of 0.1, one input layer, one output layer, and two hidden layers, leveraged a 0.3333 holdback propagation for data partitioning. This split the data into two distinct sets: a 54-run training set for error reduction and weight establishment, and a 27-run validation set for fine-tuning model selection.

**Fig. 8:**
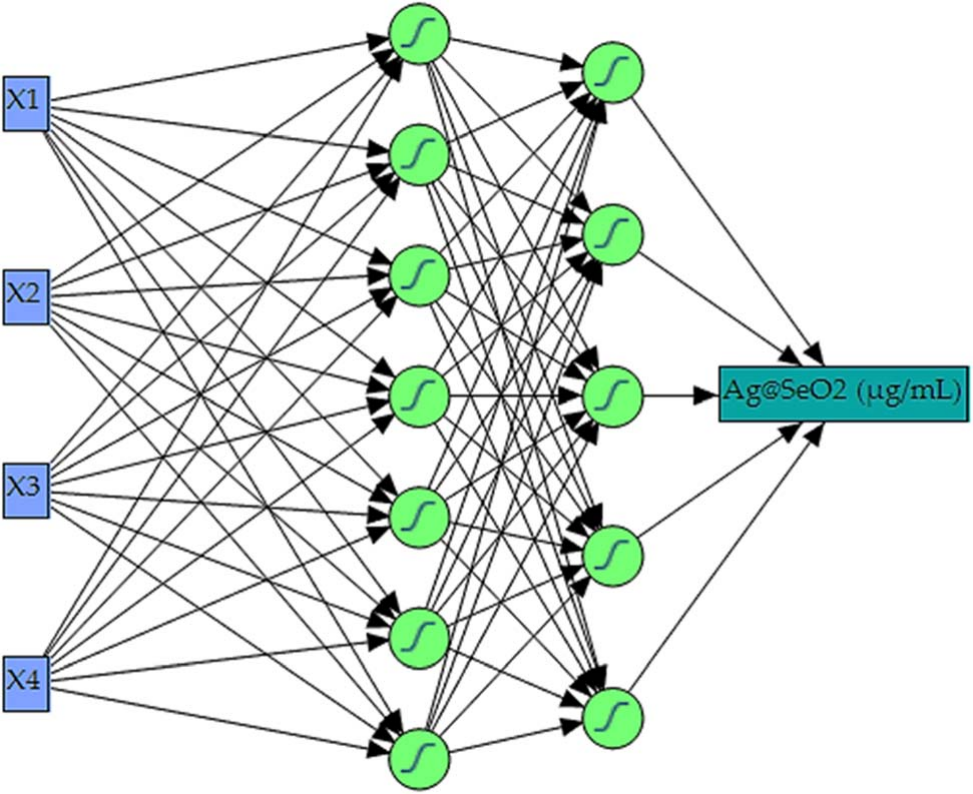
The layout ANN for Ag@SeO_2_ bmNP biosynthesis by turmeric extract. The input layer has 4 nodes (*X*_1_; pH, *X*_2_; bioconversion temperature (°C), *X*_3_; bioconversion time (h), *X*_4_; concentration of turmeric (mg mL^−1^)). The intermediate part has two layers, with 5 (first hidden layer), and 7 (second hidden layer) nodes. The output layer has only one node (Ag@SeO_2_ bmNP biosynthesis).

Within the hidden layers, all nodes utilized a shared NTanH activation function, known as, for optimized signal processing. This configuration, alongside the determined optimal network topology of 3–5–7–1 neurons (Fig. 8), facilitated superior model performance. The four input neurons directly corresponded to the investigated independent factors (pH, bioconversion temperature, time, and turmeric extract concentration). Meanwhile, the single output neuron served as the sole predictor for Ag@SeO_2_ bmNP biosynthesis. Notably, the NTanH activation function effectively supported the performance of both hidden layers, contributing to the overall model effectiveness.

Training prioritized maximizing the *R*^2^, ensuring the model closely mirrors reality. Once trained, the network's predictive power was rigorously evaluated by assessing its ability to generate outputs closely resembling, or even identical to the actual Ag@SeO_2_ bmNP values. Table 1 showcases the predicted values generated by the ANN model. Comparing them to the experimental data reveals a remarkable agreement, with the ANN outperforming the BBD model by exhibiting lower residual gaps.

The rise of artificial intelligence offers powerful tools for scientific research. Among these, ANNs stand out for their versatility and predictive prowess. Their unique architecture, employing flexible functions for input–output mapping,^69^ makes them particularly suitable for our investigation of Ag@SeO_2_ bmNP biosynthesis using turmeric extract. Unlike traditional modeling techniques, ANNs are not limited by linear relationships and can excel with the right architecture.^67^ However, unlike response surface designs, the black box nature of ANN limits the delving into complex systems of the input–output relationships.^67,70,71^ Nevertheless, the apparent mutual relationship is not crucial for our study, as the metal relationships of biological processes behind Ag@SeO_2_ bmNP biosynthesis were not our aim. Finally, ANNs can learn from diverse data types by tailoring their internal structure, *i.e.*, nodes and hidden layers.^68^ This flexibility empowers us to build an efficient model even with potentially limited or complex data.

### 3.6. Evaluation of the ANN model

#### 3.6.1. Linear relationships

**Fig. 9:**
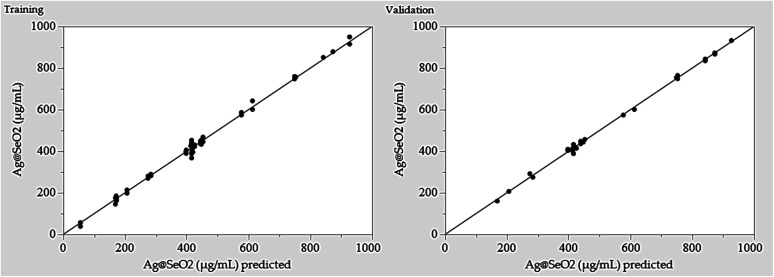
Adequacy of training and validation processes of ANN as shown by the predicted *versus* actual values of Ag@SeO_2_ bmNP biosynthesis by turmeric extract.

Model performance was rigorously assessed through both training and validation procedures. The predicted points (Fig. 9) tightly cluster around the experimental data, hugging the perfect prediction line. This close alignment in both the training and validation phases signifies two crucial things. First, the ANN faithfully mirrors reality during learning, suggesting a robust training process. Second, this remarkable alignment hints at the model's exciting potential to generalize its knowledge beyond the initial training data, a testament to its true predictive power. The BBD model lacks this generalization ability.

#### 3.6.2. Residual analysis

**Fig. 10:**
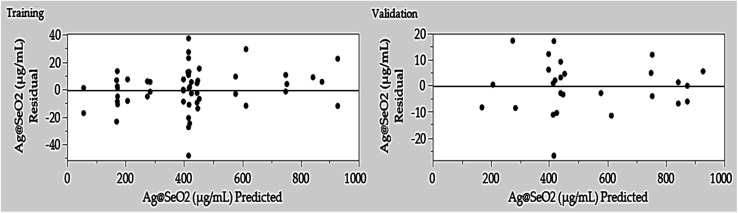
Predicted *versus* residual values of ANN for both training and validation processes of Ag@SeO_2_ bmNP biosynthesis by turmeric extract.

The residual test is usually used to assess the adequacy and fitness of training and validation processes to predict Ag@SeO_2_ bmNP biosynthesis by turmeric extract. The residual plots (Fig. 10) unveiled an evenly dispersion of the residuals, where the error values data were equally distributed around the 0-axis without linearity. The equal distribution along both sides of 0-axis indicated that the variance of Ag@SeO_2_ bmNP biosynthesis during training and validation processes was independent, verifying the adequacy and generalization capacity of the ANN model. The evenly scattered residuals, hugging the zero line without any discernible pattern, paint a picture of perfect harmony. This equal distribution on both sides of the zero axis reveals the model's predictions exhibit consistent variance, regardless of whether it's learning or validating. This lack of bias and randomness confirms the model's adequacy for its task and hints at its exciting potential to generalize its knowledge to new data, proving itself as a reliable predictor.

#### 3.6.3. Three-dimensional (3D) analysis

**Fig. 11:**
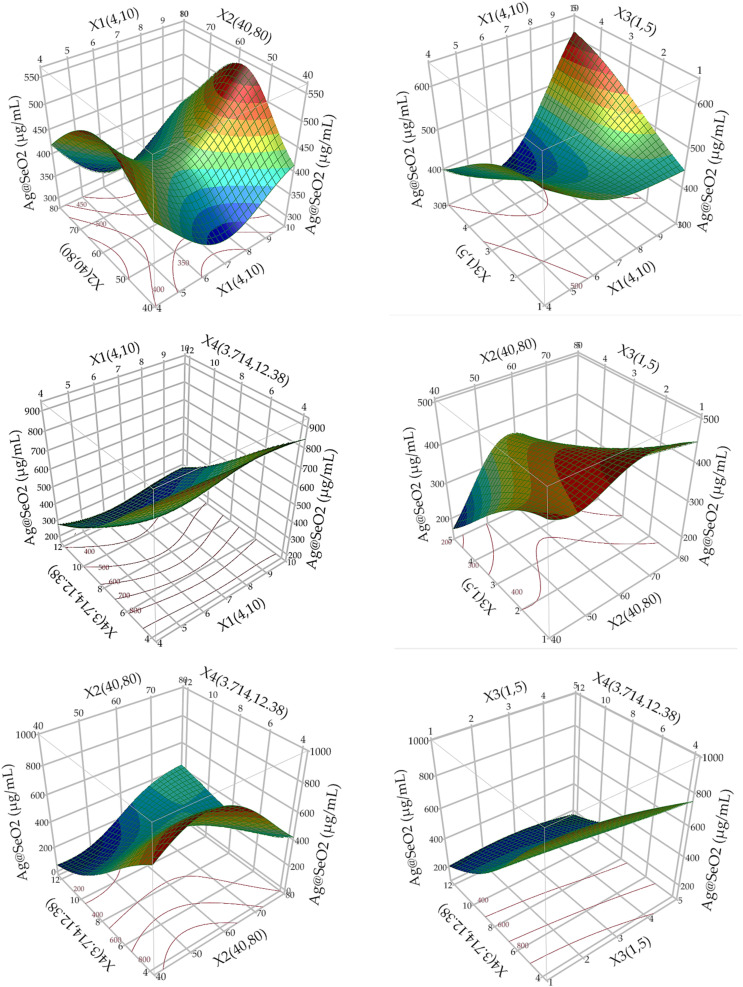
Response surface 3D plots of a pairwise pattern of the tested variables on Ag@SeO_2_ bmNP biosynthesis by turmeric extract, keeping the other variable fixed at its center level, based on ANN models. *X*_1_; pH, *X*_2_; bioconversion temperature (°C), *X*_3_; bioconversion time (h), *X*_4_; concentration of turmeric (mg mL^−1^).

A 3D surface plot analysis (Fig. 11) explored the interactions between variable pairs by holding other factors at their central levels. The plots revealed that Ag@SeO_2_ bmNP biosynthesis increased as each variable approached its optimal level, then decreased afterward. However, the high pH level and low levels of bioconversion temperature (°C), bioconversion time, and concentration of turmeric supported the bioconversion process of Ag@SeO_2_ into nano form. It is of special importance to note that Ag@SeO_2_ bmNP biosynthesis reached its peak value within the tested range and relatively near the midpoints of the design. This pattern suggests that the tested factors and their levels were sensibly chosen, and the ANN model fits the design well.^72^

The 3D plots showcased diverse patterns, this reflects ANN's proficiency in deciphering complex, nonlinear associations between inputs and outputs, regardless of their apparent relationship. Hidden layers facilitate this process, enabling indirect pathways between inputs and outputs, unlike conventional models with direct paths.^73^ This makes ANNs exceptional predictors even when input–output relationships are unclear or irrelevant.^66,70,71^

Sarkar *et al.*^74^ pointed out that the biosynthesis of silver nanoparticles occurred in pH ranging from 5.5 to 8.0, while the stability of Ag NPs was synthesized at an alkaline pH, and adequate stability was noted at pH 7. Additionally, the optimization process of Ag NPs synthesis was significantly affected by the concentration of AgNO_3_, dose of extract, and bioconversion time, whereas, the temperature of NPs synthesis did not have a significant effect. Liaqat *et al.*^75^ optimized, they found that the optimum reaction time of Ag NPs green synthesis was 60 min, the optimum temperature was 75 °C, and the optimum concentration of AgNO_3_ was 1 mM. Another study reported that the optimization process of biosynthesized AgNPs was conducted under pH 7, reaction temperature of 25 °C, 1 mM AgNO_3_ concentration, and 15–20 g of wet biomass of *Penicillium* sp.^76^ As well as, the optimized selenium nanoparticles were achieved at pH 7, and 32 °C by *Streptomyces* sp.^77^ Birla *et al.*^78^ reported the biosynthesis and optimization of silver nanoparticles at a pH range from 9 to 11 and a temperature range from 40–60 °C. As well as, they found that there are correlations between the yield of nanoparticles and the volume of filtrate, biomass quantity, and salt concentration.

### 3.7. Model performance

Initial evaluation revealed the ANN model's robust predictive capabilities, evident in minimal residuals and accurate fit to experimental data. Performance metrics calculated during training, validation, and overall stages (Table 4) confirmed this, with *R*^2^ values exceeding 0.99 and low error values (root average square error and mean absolute deviation). This demonstrates the model's high confidence and accuracy in predicting Ag@SeO_2_ bmNP biosynthesis.

**Table 4:** The statistics of training and validation stages and the overall ANN model performance for Ag@SeO_2_ bmNP biosynthesis

Measure	Training	Validation	Overall model
*R* ^2^	0.9953	0.9980	0.9965
Root average square error	14.687	9.549	13.198
Mean absolute deviation	10.876	7.401	9.718
Sum frequency	54	27	81

### 3.8. Optimum conditions and model validation

**Fig. 12:**
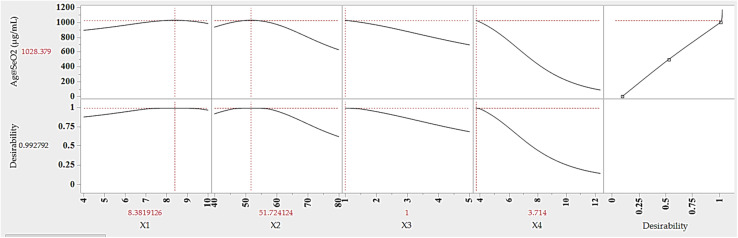
Desirability function, and prediction value of Ag@SeO_2_ bmNP biosynthesis by turmeric extract under the optimum conditions determined by ANN model. *X*_1_; pH, *X*_2_; bioconversion temperature (°C), *X*_3_; bioconversion time (h), *X*_4_; concentration of turmeric (mg mL^−1^).

The ANN model was used to predict the optimum levels of the four tested factors that maximize Ag@SeO_2_ bmNP biosynthesis conditions (Fig. 12). The calculated values were found to be 9.83 pH, 51.7 °C bioconversion temperature, 1.0 h bioconversion time, and 3.71 mg mL^−1^ of turmeric extract. Under such conditions, the response of Ag@SeO_2_ bmNP biosynthesis was calculated to be 1028.38 μg mL^−1^, with a desirability value of 0.9928. Subsequently, the estimated optimum conditions for the 4 variables were verified through laboratory experimentation. The validation test revealed high reliability with the actual Ag@SeO_2_ bmNP biosynthesis yield, being 1034.53 ± 17.38 μg mL^−1^, exhibiting high performance of the ANN model.

To pinpoint the ideal conditions for maximizing Ag@SeO_2_ bmNP biosynthesis, the desirability function was used. These versatile tools act as guides, mathematically evaluating different scenarios and assigning scores ranging from 0 (undesirable) to 1 (highly desirable), this is considered a crucial step before model validation to set clear targets for prediction accuracy.^28,64,79^ The predicted values aligned remarkably with the experimental findings, affirming the desirability function's success in guiding us toward optimal conditions for biosynthesis.

Ruling the realm of complex systems, ANNs are the undisputed navigators, effortlessly unraveling riddles of nonlinearity and multicollinearity compared with the other models. With their intricate internal compass, they chart hidden pathways and uncover cryptic connections, revealing secrets that simpler models could never do. While other models might take a direct path to the target, ANNs embrace a more intricate journey, weaving through hidden layers and iterations to unlock hidden patterns.^28,29^ This meticulous approach yields impressive predictive accuracy, but it also comes with trade-offs. The process demands time for modeling, and the intricate connections can make it challenging to pinpoint the exact role of individual factors within the model.^71,80^ This complexity can sometimes limit the ability to simplify the model or isolate individual factors for further exploration.

### 3.9. Phytochemical analyses

**Fig. 13:**
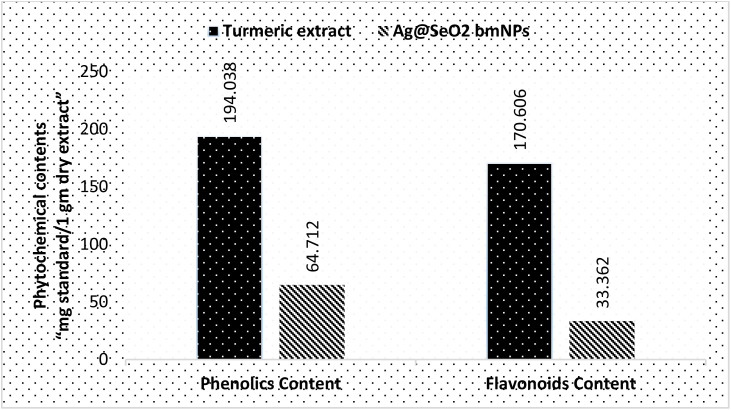
Comparison of the phytochemical contents of turmeric extract and Ag@SeO_2_ bmNPs.

The phytochemical analyses (Fig. 13 and Table S4†) revealed that turmeric extract has a significantly higher phenolics content (194.038 ± 2.8 mg g^−1^) compared to Ag@SeO_2_ bmNPs (64.712 ± 1.3 mg g^−1^). This suggests that turmeric extract is a richer source of phenolic compounds, which are known to have antioxidant and other health-promoting properties. Similar to phenolics content, turmeric extract also has a higher flavonoid content (170.606 ± 0.59 mg g^−1^) compared to Ag@SeO_2_ bmNPs (33.362 ± 0.74 mg g^−1^). The high content of phenolics and flavonoids in the turmeric extract indicates the potential for strong antioxidant activity. Based on the literature reports, turmeric extract is a rich source of phenolic content such as curcuminoids (*e.g.*, curcumin, dimethoxy-curcumin, and bis-dimethoxy-curcumin),^81,82^ which are the most abundant phenolic compounds in turmeric, responsible for its characteristic yellow color. In addition, turmeric extract includes other types of phenolic contents such as ferulic acid benefits cardiovascular and skin health, vanillin “contributes to the pleasant flavor of turmeric”, and *p*-coumaric acid “phenolic acid”. Turmeric extract on the other side also is a rich source of flavonoid contents which contributes to its antioxidant activity such as quercetin, kaempferol, gallic acid, and naringenin cholesterol-lowering effects.^83,84^

Phytochemicals *e.g.* phenolic compounds like curcumin readily donate electrons to metal ions, triggering their reduction to MNPs.^85^ These electron-rich molecules act as mild and biocompatible alternatives to harsh chemical-reducing agents used in conventional synthesis.^86^ Phytochemicals also prevent the newly formed MNPs from aggregating and growing uncontrollably. Phytochemicals often possess functional groups like hydroxyl or amine groups that bind to the MNP surface, providing steric hindrance and electrostatic repulsion.^87^ This capping ability ensures size control and stability, crucial for desired MNP properties. Phytochemicals influence the size, shape, and morphology of the resulting MNPs.^88^ Specific interactions between phytochemicals and metal ions can direct the nucleation and growth process, leading to MNPs with tailored properties for specific applications.

### 3.10. Antioxidant activity

**Fig. 14:**
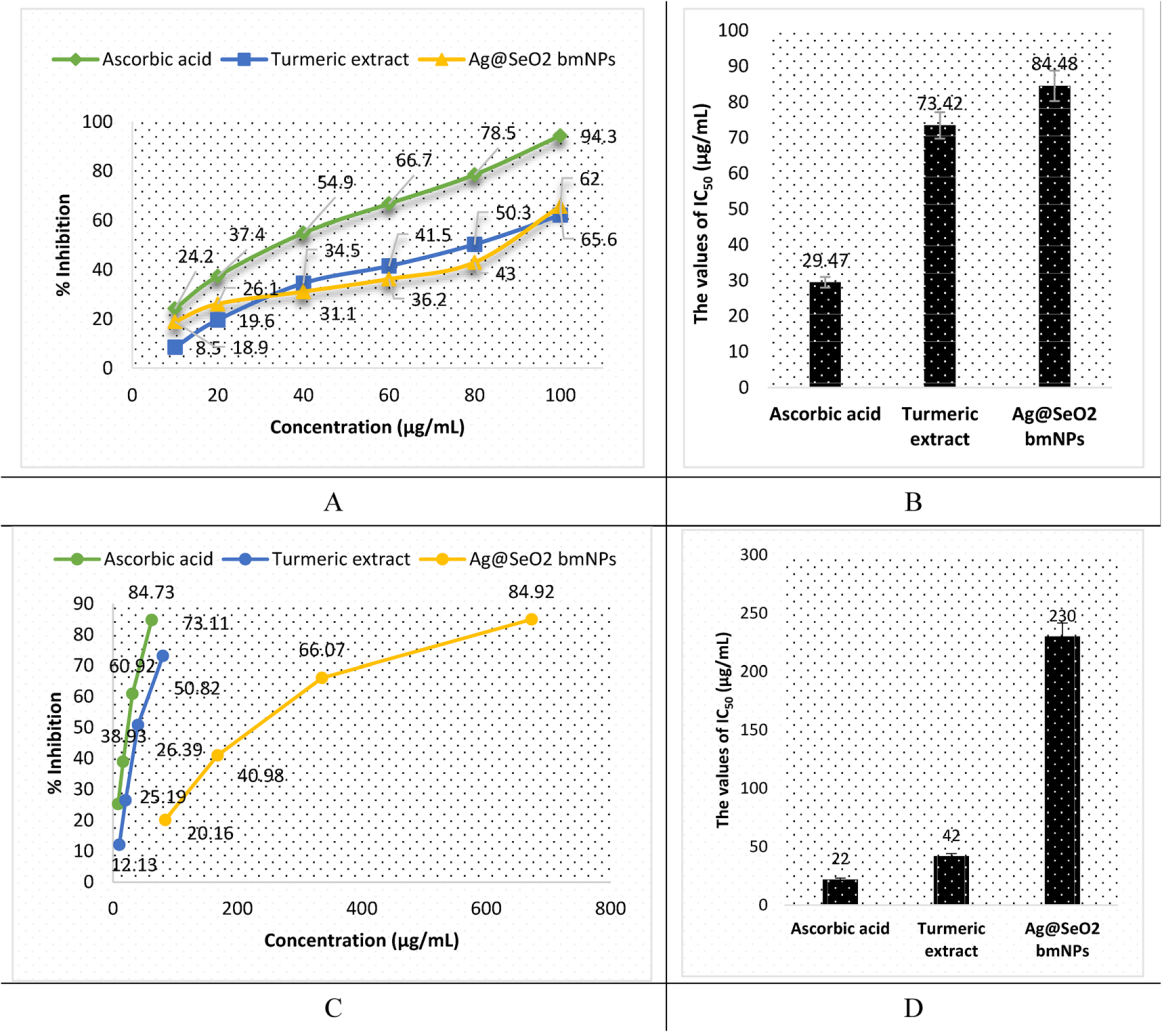
The results of antioxidant activity by ABTS assay. (A) and (C) refer to the relationship between the sample concentration and % of scavenging activity for the results obtained by ABTS and DPPH assays, respectively. (B) and (D) refer to the antioxidant capacity expressed as IC_50_ values for the results obtained by ABTS and DPPH assays, respectively.

The antioxidant activity of turmeric extract, Ag mNPs, and Ag@SeO_2_ bmNPs was assessed by ABTS assay. The results (Fig. 14A, B and Table S5†) showed that turmeric extract and Ag@SeO_2_ bmNPs have antioxidant activity, but turmeric extract is the most potent antioxidant. The IC_50_ values for turmeric extract, Ag@SeO_2_ bmNPs, and ascorbic acid are 29.47, 73.42, and 84.48 μg mL^−1^, respectively. This means that turmeric extract can inhibit the ABTS radical by 50% at a concentration that is lower than the concentration of Ag@SeO_2_ bmNPs, or ascorbic acid. The antioxidant activity of turmeric extract is likely due to the presence of curcuminoids, which are polyphenols with strong antioxidant properties. Curcuminoids have been shown to scavenge free radicals, chelate metal ions, and inhibit the formation of reactive oxygen species (ROS). The antioxidant activity of Ag@SeO_2_ bmNPs is likely due to the presence of silver nanoparticles, which have been shown to scavenge free radicals and inhibit the formation of ROS. Ag@SeO_2_ bmNPs may also have enhanced antioxidant activity due to the presence of selenium, which is an essential nutrient with antioxidant properties.

Briefly, the results of the ABTS assay show that turmeric extract, and Ag@SeO_2_ bmNPs all have antioxidant activity, but turmeric extract is the most potent antioxidant. This suggests that turmeric extract may be a useful dietary supplement or ingredient in functional foods for the prevention of oxidative stress and related diseases. In addition, the antioxidant activity of turmeric extract, and Ag@SeO_2_ bmNPs increases with increasing concentration. This is expected, as antioxidants typically scavenge free radicals in a dose-dependent manner. The antioxidant activity of turmeric extract is comparable to that of ascorbic acid, which is a known antioxidant. This suggests that turmeric extract may be an effective alternative to ascorbic acid for preventing oxidative stress and related diseases. The results of this study are promising and suggest that turmeric extract and Ag@SeO_2_ bmNPs may have the potential as antioxidants for the prevention and treatment of oxidative stress-related diseases.

The antioxidant activity by DPPH assay is shown in Fig. 14C, D and Table S6.† Turmeric extract demonstrates potent antioxidant activity, suggesting strong free radical scavenging potential. The IC_50_ value of 42 μg mL^−1^ of turmeric extract indicates that it is comparable and effective to ascorbic acid (IC_50_ = 22 μg mL^−1^) in this specific assay. In addition, turmeric extract shows strong dose-dependent antioxidant activity, scavenging up to 73.11% of DPPH radicals at the lowest concentration (80 μg mL^−1^). However, Ag@SeO_2_ bmNPs revealed lower antioxidant activity than the turmeric extract, as reflected by the higher IC_50_ value (230 μg mL^−1^). Ag@SeO_2_ bmNPs displayed weaker antioxidant activity compared to turmeric extract at all concentrations and showed a dose-dependent increase in DPPH scavenging but reached only 84.92% at the highest concentration (673 μg mL^−1^). Both ABTS and DPPH assays show that turmeric extract exhibits strong antioxidant activity compared to Ag@SeO_2_ bmNPs.

The mechanism of action was illustrated as the turmeric extract contains curcuminoids, which are polyphenols with strong antioxidant properties.^89^ Curcuminoids can scavenge free radicals, such as superoxide radicals, hydroxyl radicals, and peroxyl radicals, by donating hydrogen atoms or electrons. Turmeric extract contains curcuminoids, which can chelate metal ions and prevent them from catalyzing free radical formation.^90^ ROS, such as superoxide radicals and hydrogen peroxide, are produced during normal cellular metabolism. However, excessive ROS production can lead to oxidative stress and cell damage. Curcuminoids can inhibit the formation of ROS by inhibiting enzymes that produce ROS, such as NADPH oxidase and xanthine oxidase.^91^ There are a few reasons why nanoparticles may be less potent antioxidants than turmeric extract: the size and shape of nanoparticles can affect their antioxidant activity. Smaller nanoparticles have a larger surface area, which allows them to interact with and scavenge free radicals more efficiently.^92^ However, nanoparticles can also agglomerate, which can reduce their surface area and antioxidant activity.

Turmeric extract contains a variety of curcuminoids, which have different sizes and shapes. This variety of sizes and shapes may allow curcuminoids to interact with and scavenge a wider range of free radicals. The surface chemistry of nanoparticles can also affect their antioxidant activity. Nanoparticles with a positively charged surface tend to be more potent antioxidants than nanoparticles with a negatively charged surface.^93^ This is because positively charged nanoparticles can attract and scavenge free radicals more efficiently. Nanoparticles can interact with biological systems in complex ways.^94^ Some nanoparticles can be toxic to cells, while others can be taken up by cells and metabolized. The biocompatibility of nanoparticles can affect their antioxidant activity. For example, if nanoparticles are toxic to cells, they may damage cells and generate oxidative stress.^95^ This would reduce the antioxidant activity of the nanoparticles. Turmeric extract is generally well-tolerated by humans and has a low toxicity profile. This makes it a more biocompatible antioxidant than nanoparticles.

### 3.11. Antibacterial activity

#### 3.11.1. Agar well-diffusion assay

**Fig. 15:**
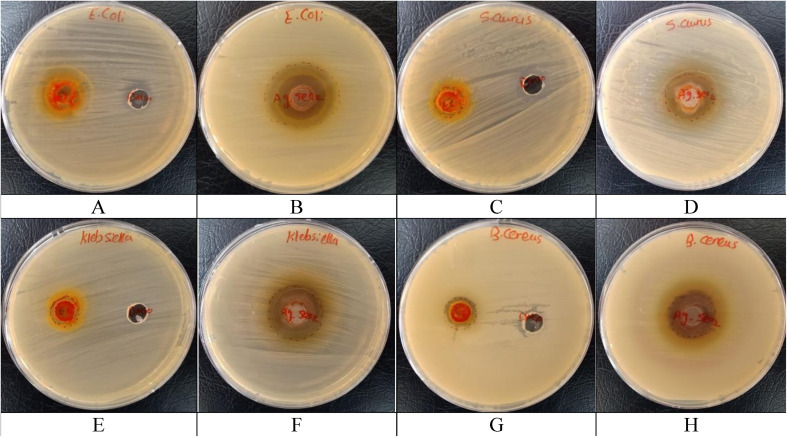
The images of Petri dishes of bacteria showing zones of inhibition around antibacterial wells in a well-diffusion assay. (A) and (B) expressed *E. coli* species; (C) and (D) expressed *S. aureus* species; (E) and (F) expressed *K. pneumoniae* species; (G) and (H) expressed *B. cereus* species. (A), (C), (E) and (G) turmeric extract and DMSO (negative control). (B), (D), (F) and (H) expressed bacterial species treated with Ag@SeO_2_ bmNPs.

The antibacterial activity of turmeric extract and Ag@SeO_2_ bmNPs was evaluated by a well-diffusion assay against *E. coli*, *S. aureus*, *K. pneumoniae*, and *B. cereus*. The results in Table 5 and Fig. 15 suggest a promising antibacterial activity for both turmeric extract and Ag@SeO_2_ bmNPs against four common bacterial strains. Turmeric extract shows moderate activity against all four strains, with inhibition zone diameters ranging from 13 to 14 mm. The plant extract does not surpass Cefotaxime in activity against *E. coli* and *S. aureus* but exhibits comparable efficacy against *K. pneumoniae* and *B. cereus*. On the other hand, Ag@SeO_2_ bmNPs demonstrate potent activity against all strains, with inhibition zones ranging from 22 to 32 mm. Ag@SeO_2_ bmNPs significantly outperform both turmeric extract and Cefotaxime against *E. coli* and *S. aureus*, suggesting potentially broad-spectrum and potent activity. In addition, Ag@SeO_2_ bmNPs show similar efficacy to Cefotaxime against *K. pneumoniae* and slightly lower against *B. cereus*.

**Table 5:** Antibacterial activity of turmeric extract and Ag@SeO_2_ bmNPs against common bacterial strains

Samples	Inhibition zone diameters (mm)			
	*E. coli* (ATCC 10536)	*S. aureus* (ATCC 6538)	*K. pneumoniae* (ATCC 10031)	*B. cereus* (EMCC number 1080)
Turmeric extract	−ve	13 ± 1.61	13 ± 1.19	14 ± 2.06
Ag@SeO_2_ bmNPs	32 ± 1.25	22 ± 0.79	25 ± 0.82	22 ± 1.03
Control (DMSO)	−ve	−ve	−ve	−ve
Cefotaxime	23 ± 0.95	11 ± 2.50	25 ± 1.74	20 ± 1.96

The moderate and broad-spectrum activity of turmeric extract suggests a multi-target or non-specific mechanism of action. The possible mechanism of action could be suggested as curcumin, a major component of turmeric, is known to interact with bacterial membranes, potentially causing leakage of essential components and cell death.^96^ Curcumin and other bioactive compounds in the extract might inhibit key enzymes involved in bacterial metabolism or protein synthesis, hindering their growth and survival.^97^ Curcumin exhibits antioxidant properties that could scavenge free radicals generated by bacteria, causing oxidative stress and damage.^98^ Turmeric extract could potentially interfere with bacterial communication pathways, disrupting their ability to coordinate defense mechanisms or biofilm formation.^99^

The potent and broad-spectrum activity of Ag@SeO_2_ bmNPs points towards a more direct and efficient mechanism of action, likely involving silver ions released from the nanoparticles. The mechanism of action of Ag@SeO_2_ bmNPs was commended as follows: silver ions bind to thiol groups on bacterial proteins and membranes, causing disruption and leakage of essential components.^100^ This explains the potent and broad-spectrum activity of Ag@SeO_2_ bmNPs. Silver ions can induce the production of ROS within bacteria, causing oxidative stress and damage to DNA, proteins, and other cellular structures.^101^ Silver ions can bind to bacterial DNA, inhibiting its replication and preventing cell division.^102^

El-deeb *et al.*^77^ found that Ag NPs have potential activity towards *E. coli* ranging 13–20 mm inhibition zone under different pH values. Kim *et al.*^103^ showed a similar effect of Ag NPs against *E. coli* and *S. aureus*. Singh *et al.*^76^ found the antibacterial activity of Ag NPs against MDR *E. coli* and *S. aureus* being 17 and 16 mm inhibition zones, respectively. Ninganagouda *et al.*^104^ reported that the Ag NPs showed potentiality against *E. coli*. The SeO_2_ coating on the nanoparticles might enhance the antibacterial activity by generating additional ROS or acting as a slow-release reservoir for silver ions.^105^ Frequently, the results presented in Table 4 warrant further investigation of both turmeric extract and Ag@SeO_2_ bmNPs as potential alternatives or supplements to Cefotaxime. Their diverse activities and promising initial results encourage continued research to elucidate their mechanisms of action, optimize their effectiveness, and explore their potential for clinical applications.

#### 3.11.2. Minimum inhibitory concentration (MIC)

The data presented in Tables 6, S7–S10 and Fig. S2† shows the MICs of tested Ag@SeO_2_ bmNPs against four bacterial strains: *B. cereus*, *K. pneumoniae*, *E. coli*, and *S. aureus*. The O.D. 600 readings at the MICs are also provided, indicating the level of bacterial growth inhibition at those concentrations. All four bacterial strains were susceptible to Ag@SeO_2_ bmNPs, with MICs ranging from 165.625 μg mL^−1^ to 331.25 μg mL^−1^. *B. cereus* and *K. pneumoniae* showed slightly higher MICs (331.25 μg mL^−1^) compared to *E. coli* and *S. aureus* (165.625 μg mL^−1^). This suggests a slightly lower susceptibility of these two strains to Ag@SeO_2_ bmNPs. O.D. 600 readings at the MICs were close to zero for all strains, indicating minimal or no detectable bacterial growth at these concentrations. This highlights the effectiveness of the Ag@SeO_2_ bmNPs in inhibiting bacterial growth. The lower MICs for *E. coli* and *S. aureus* suggest they might be more susceptible to the mode of action of Ag@SeO_2_ bmNPs compared to *B. cereus* and *K. pneumoniae* species.

**Table 6:** MIC values (μg mL^−1^) and corresponding O.D. 600 readings for bacterial growth inhibition by treatment with Ag@SeO_2_ bmNPs

Bacteria strain	MIC (μg mL^−1^)	O.D. 600 at MIC
*B. cereus*	331.25	0.007
*K. pneumoniae*	331.25	0.001
*E. coli*	165.625	0.001
*S. aureus*	165.625	0.001

## 4. Conclusion

Phytochemicals in turmeric extract perform a crucial role in reducing SeO_2_ into Ag@SeO_2_ bmNPs. Their electron-donating capabilities drive the reduction, while their functional groups contribute to nanoparticle stabilization and shape control. This green and sustainable approach overlays the way for successful biosynthesis of Ag@SeO_2_ bmNPs, with various applications and desirable characteristics. Ag@SeO_2_ bmNPs are characterized by their negative surface charge, spherical morphology, crystalline nature, core–shell structure, and consistent elemental composition. Combining BBD and ANN modeling effectively optimizes Ag@SeO_2_ bmNP biosynthesis using turmeric extract. ANN demonstrated superior predictive accuracy and identified optimal conditions for maximizing yield. However, its black-box nature necessitates further investigation into individual factor effects and exploration of alternative modeling approaches for deeper mechanistic understanding and process refinement. Turmeric extract is a rich source of bioactive compounds with potent antioxidant and antibacterial properties. Bio-synthesized Ag@SeO_2_ bmNPs show promising antioxidants and their potential activity against various bacterial strains. Ultimately, further investigation is needed to elucidate the exact mechanisms of action and optimize the effectiveness of both turmeric extract and bmNPs for potential applications in food preservation, medicine, and other fields.

## Conflicts of interest

The authors declare no conflict of interest.

## Supplementary Material

RA-014-D4RA00004H-s001

## References

[cit1] Sharma D., Kanchi S., Bisetty K. (2019). Arabian J. Chem..

[cit2] Soni V., Raizada P., Singh P., Cuong H. N., Rangabhashiyam S., Saini A., Saini R. V., Van Le Q., Nadda A. K., Le T. T., Nguyen V. H. (2021). Environ. Res..

[cit3] Amalraj A., Pius A., Gopi S., Gopi S. (2017). J. Tradit. Complementary Med..

[cit4] Chattopadhyay I., Biswas K., Bandyopadhyay U., Banerjee R. K. (2004). Curr. Sci..

[cit5] Sharifi-Rad J., Rayess Y. E., Rizk A. A., Sadaka C., Zgheib R., Zam W., Sestito S., Rapposelli S., Neffe-Skocińska K., Zielińska D., Salehi B. (2020). Front. Pharmacol.

[cit6] Verma R. K., Kumari P., Maurya R. K., Kumar V., Verma R. B., Singh R. K. (2018). Int. J. Chem. Stud..

[cit7] Jaiswal S., Mishra P. (2018). Med. Microbiol. Immunol..

[cit8] Chhabria S., Desai K. (2016). Encycl. Nanosci. Nanotechnol..

[cit9] Ali M. Y., Abad W. K., Roomy H. M., Abd A. N. (2023). NanoWorld J..

[cit10] Sarkar R. D., Kalita M. C. (2022). Heliyon.

[cit11] Medina-Cruz D. , Saleh B., Vernet-Crua A., Nieto-Argüello A., Lomelí-Marroquín D., Vélez-Escamilla L. Y., Cholula-Díaz J. L., García-Martín J. M. and Webster T., Racing for the Surface: Antimicrobial and Interface Tissue Engineering, Springer, Cham, 2020, pp. 397–434, 10.1007/978-3-030-34471-9_16

[cit12] Ali I. , Peng C., Naz I., Amjed M. A., Magnetic Nanostructures: Environmental and Agricultural Applications, 2019, pp. 161–179

[cit13] Sári D., Ferroudj A., Dávid S., El-Ramady H., Abowaly M., Abdalla Z. F., Mansour H., Eid Y., Prokisch J. (2024). Egypt. J. Soil Sci..

[cit14] Din M. I., Rani A. (2018). Crit. Rev. Anal. Chem..

[cit15] Soni V., Raizada P., Singh P., Cuong H. N., Rangabhashiyam S., Saini A., Saini R. V., Van Le Q., Nadda A. K., Le T. T., Nguyen V. H. (2021). Environ. Res..

[cit16] Saravanan M. , Barabadi H. and Vahidi H., Green nanotechnology: isolation of bioactive molecules and modified approach of biosynthesis, in Biogenic Nanoparticles for Cancer Theranostics, Elsevier, 2021, pp. 101–122

[cit17] Cuenya B. R. (2010). Thin Solid Films.

[cit18] Zhang R., Khalizov A., Wang L., Hu M., Xu W. (2012). Chem. Rev..

[cit19] Karthikeyan S., Ramamoorthy B. (2014). Appl. Surf. Sci..

[cit20] Javed R., Zia M., Naz S., Aisida S. O., Ain N. U., Ao Q. (2020). J. Nanobiotechnol..

[cit21] Kowalczyk B., Bishop K. J., Lagzi I., Wang D., Wei Y., Han S., Grzybowski B. A. (2012). Nat. Mater..

[cit22] Østergård T., Jensen R. L., Mikkelsen F. S. (2020). Energy Build..

[cit23] Jankovic A., Chaudhary G., Goia F. (2021). Energy Build..

[cit24] Tao H., Wu T., Aldeghi M., Wu T. C., Aspuru-Guzik A., Kumacheva E. (2021). Nat. Rev. Mater..

[cit25] Sun Y. (2013). Chem. Soc. Rev..

[cit26] Salem S. S., Fouda A. (2021). Biol. Trace Elem. Res..

[cit27] Yang X. Y., Chen L. H., Li Y., Rooke J. C., Sanchez C., Su B. L. (2017). Chem. Soc. Rev..

[cit28] El-Metwally M. M., Abdel-Fattah G. M., Al-Otibi F. O., Khatieb D. K. E., Helmy Y. A., Mohammed Y. M., Saber W. I. (2023). Heliyon.

[cit29] Saber W. I. A., Ghoniem A. A., Al-Otibi F. O., El-Hersh M. S., Eldadamony N. M., Menaa F., Elattar K. M. (2023). Sci. Rep..

[cit30] Arawande J. O., Akinnusotu A., Alademeyin J. O. (2018). Int. J. Tradit. Nat. Med..

[cit31] Himesh S., Sharan P. S., Mishra K., Govind N., Singhai A. K. (2011). Int. Res. J. Pharm..

[cit32] Amalraj A., Pius A., Gopi S., Gopi S. (2017). J. Tradit. Complementary Med..

[cit33] Jayaprakasha G. K., Jagan Mohan Rao L., Sakariah K. K. (2002). J. Agric. Food Chem..

[cit34] Ahmed T., Gilani A. H. (2014). Phytother. Res..

[cit35] Madhusankha G. D. M. P., Siow L. F., Thoo Y. Y. (2023). Food Biosci..

[cit36] Umar N. M., Parumasivam T., Aminu N., Toh S. M. (2020). J. Appl. Pharm. Sci..

[cit37] de Oliveira Filho J. G. , de Almeida M. J., Sousa T. L., dos Santos D. C. and Egea M. B., Bioactive Compounds in Underutilized Vegetables and Legumes, 2021, pp. 297–318

[cit38] Himesh S., Sharan P. S., Mishra K., Govind N., Singhai A. K. (2011). Int. Res. J. Pharm..

[cit39] Labban L. (2014). Int. J. Pharm. Biomed. Sci..

[cit40] Bhat A., Mahalakshmi A. M., Ray B., Tuladhar S., Hediyal T. A., Manthiannem E., Padamati J., Chandra R., Chidambaram S. B., Sakharkar M. K. (2019). BioFactors.

[cit41] Wongcharoen W., Phrommintikul A. (2009). Int. J. Cardiol..

[cit42] Akaberi M. , Sahebkar A. and Emami S. A., Studies on Biomarkers and New Targets in Aging Research in Iran: Focus on Turmeric and Curcumin, 2021, pp. 15–39

[cit43] Van Nong H., Hung L. X., Thang P. N., Chinh V. D., Vu L. V., Dung P. T., Van Trung T., Nga P. T. (2016). SpringerPlus.

[cit44] Elattar K. M., Ghoniem A. A., Al-Otibi F. O., El-Hersh M. S., Helmy Y. A., Saber W. I. (2023). Appl. Sci..

[cit45] Blainski A., Lopes G. C., De Mello J. C. P. (2013). Molecules.

[cit46] Mammen D., Daniel M. (2012). Food Chem..

[cit47] Miller N. J., Rice-Evans C. A. (1997). Free Radical Res..

[cit48] Mensor L. L., Menezes F. N., Leitao L. C., Reis A. S., Santos T. C., Coube C. S., Oliveira A. G. (2001). Phytomedicine.

[cit49] Mphande I., Kataba A., Muzandu K., Gono-Bwalya A. (2022). J. Evidence-Based Complementary Altern. Med..

[cit50] Parvekar P., Palaskar J., Metgud S., Maria R., Dutta S. (2020). Biomater. Invest. Dent..

[cit51] Sharma C., Ansari S., Ansari M. S., Satsangee S. P. (2022). J. Ind. Eng. Chem..

[cit52] El-Seedi H. R., El-Shabasy R. M., Khalifa S. A., Saeed A., Shah A., Shah R., Iftikhar F. J., Abdel-Daim M. M., Omri A., Hajrahand N. H., Sabir J. S. (2019). RSC Adv..

[cit53] Cho W. S., Thielbeer F., Duffin R., Johansson E. M., Megson I. L., MacNee W., Bradley M., Donaldson K. (2014). Nanotoxicology.

[cit54] Zhu M., Wang H., Keller A. A., Wang T., Li F. (2014). Sci. Total Environ..

[cit55] Luo D., Yan C., Wang T. (2015). Small.

[cit56] Zhang H., Banfield J. F. (2005). Chem. Mater..

[cit57] Guo Y. G., Lee J. S., Maier J. (2006). Solid State Ionics.

[cit58] Park Y. S., Dmytruk A., Dmitruk I., Kasuya A., Okamoto Y., Kaji N., Tokeshi M., Baba Y. (2010). J. Phys. Chem. C.

[cit59] Cartwright A., Jackson K., Morgan C., Anderson A., Britt D. W. (2020). Agronomy.

[cit60] Al-Asfar A., Zaheer Z., Aazam E. S. (2018). J. Photochem. Photobiol., B.

[cit61] Im S. W., Ahn H. Y., Kim R. M., Cho N. H., Kim H., Lim Y. C., Lee H. E., Nam K. T. (2020). Adv. Mater..

[cit62] Zhang Q., Yang X., Ji H., Sun D., Liu Y. (2013). ACS Appl. Mater. Interfaces.

[cit63] Xu H., Wang F., Zhu Y. (2012). J. Colloid Interface Sci..

[cit64] El-Hersh M. S., Saber W., El-Fadaly H. A., Mahmoud M. K. (2016). Pak. J. Biol. Sci..

[cit65] Lau H.-L., Wong F. W. F., Abd Rahman R. N. Z. R., Mohamed M. S., Ariff A. B., Hii S.-L. (2023). Biocatal. Agric. Biotechnol..

[cit66] Elsayed A., Moussa Z., Alrdahe S. S., Alharbi M. M., Ghoniem A. A., El-Khateeb A. Y., Saber W. I. (2022). Front. Microbiol..

[cit67] Pandiselvam R., Prithviraj V., Manikantan M., Beegum P. S., Ramesh S., Padmanabhan S., Kothakota A., Mathew A., Hebbar K., Khaneghah A. M. (2022). Measurement: Food.

[cit68] Srikanth V., Rajesh G., Kothakota A., Pandiselvam R., Sagarika N., Manikantan M., Sudheer K. (2020). Computers and Electronics in Agriculture.

[cit69] Beier K. T., Steinberg E. E., DeLoach K. E., Xie S., Miyamichi K., Schwarz L., Gao X. J., Kremer E. J., Malenka R. C., Luo L. (2015). Cell.

[cit70] Moussa Z., Darwish D. B., Alrdahe S. S., Saber W. I. (2021). Front. Microbiol..

[cit71] Saber W. I., El-Naggar N. E.-A., El-Hersh M. S., El-Khateeb A. Y., Elsayed A., Eldadamony N. M., Ghoniem A. A. (2021). Sci. Rep..

[cit72] Al-Askar A. A., Rashad E. M., Moussa Z., Ghoneem K. M., Mostafa A. A., Al-Otibi F. O., Arishi A. A., Saber W. I. (2022). Front. Microbiol..

[cit73] Anjaly M. G., Prince M., Warrier A. S., Lal A. N., Mahanti N. K., Pandiselvam R., Thirumdas R., Sreeja R., Rusu A. V., Trif M. (2022). Ultrason. Sonochem..

[cit74] Sarkar M., Denrah S., Das M., Das M. (2021). Chemical Physics Impact.

[cit75] Liaqat N., Jahan N., Anwar T., Qureshi H. (2022). Front. Chem..

[cit76] Singh D., Rathod V., Ninganagouda S., Hiremath J., Singh A. K., Mathew J. (2014). Bioinorg. Chem. Appl..

[cit77] El-deeb B. A., Asem E., Mohammed K. (2023). Sohag Journal of Sciences.

[cit78] Birla S. S., Gaikwad S. C., Gade A. K., Rai M. K. (2013). Sci. World J..

[cit79] Saber W. I., El-Naggar N. E., El-Hersh M. S., El-Khateeb A. Y. (2015). Biotechnology.

[cit80] Elsayed M. S., Eldadamony N. M., Alrdahe S. S., Saber W. I. (2021). Molecules.

[cit81] E Wright L., Frye J. B., Gorti B., Timmermann B. N., Funk J. L. (2013). Curr. Pharm. Des..

[cit82] Jacob J. N. (2016). Stud. Nat. Prod. Chem..

[cit83] Arawande J. O., Akinnusotu A., Alademeyin J. O. (2018). Int. J. Tradit. Nat. Med..

[cit84] Jing L. J., Mohamed M., Rahmat A., Bakar M. F. A. (2010). J. Med. Plants Res..

[cit85] Priyadarsini K. I. (2014). Molecules.

[cit86] Arcadi A. (2008). Chem. Rev..

[cit87] Hariharan K., Patel P., Mehta T. (2022). Pharm. Dev. Technol..

[cit88] Phukan S., Bharali P., Das A. K., Rashid M. H. (2016). RSC Adv..

[cit89] Lim H. S., Park S. H., Ghafoor K., Hwang S. Y., Park J. (2011). Food Chem..

[cit90] Amalraj A., Pius A., Gopi S., Gopi S. (2017). J. Tradit. Complementary Med..

[cit91] Yousefian M., Shakour N., Hosseinzadeh H., Hayes A. W., Hadizadeh F., Karimi G. (2019). Phytomedicine.

[cit92] Xue Y., Luan Q., Yang D., Yao X., Zhou K. (2011). J. Phys. Chem. C.

[cit93] Patil S., Sandberg A., Heckert E., Self W., Seal S. (2007). Biomaterials.

[cit94] Mu Q., Jiang G., Chen L., Zhou H., Fourches D., Tropsha A., Yan B. (2014). Chem. Rev..

[cit95] Horie M., Tabei Y. (2021). Free Radical Res..

[cit96] Zheng D., Huang C., Huang H., Zhao Y., Khan M. R. U., Zhao H., Huang L. (2020). Chem. Biodiversity.

[cit97] Gul F. Z., Basheer M. (2016). J. Ayurvedic Herb. Med..

[cit98] Ak T., Gülçin I. (2008). Chem.-Biol. Interact..

[cit99] Nazzaro F. , Fratianni F., d'Acierno A., De Feo V., Ayala-Zavala F. J., Gomes-Cruz A., Granato D. and Coppola R., Effect of polyphenols on microbial cell-cell communications, in Quorum Sensing, Academic Press, 2019, pp. 195–223

[cit100] Khalandi B., Asadi N., Milani M., Davaran S., Abadi A. J. N., Abasi E., Akbarzadeh A. (2017). Drug Res..

[cit101] Flores-López L. Z., Espinoza-Gómez H., Somanathan R. (2019). J. Appl. Toxicol..

[cit102] Khalandi B., Asadi N., Milani M., Davaran S., Abadi A. J. N., Abasi E., Akbarzadeh A. (2017). Drug Res..

[cit103] Kim J. S., Kuk E., Yu K. N., Kim J. H., Park S. J., Lee H. J., Kim S. H., Park Y. K., Park Y. H., Hwang C. Y., Kim Y. K. (2007). Nanomedicine.

[cit104] Ninganagouda S., Rathod V., Jyoti H., Singh D., Prema K., Haq M. U. (2013). Int. J. Pharma Bio Sci..

[cit105] Murthy P. S. , Das A., Ramalingam B., Sekar R. and Dobretsov S., Nanotechnology Approaches to Treating Antimicrobial Resistant Infections Caused by Biofilms: Research Needs for Translation from In Vitro to In Vivo Applications, in Biofilm Associated Antimicrobial Resistance and Its Recovery, CRC Press, 2013, pp. 116–158

