# Supplementary material for: Turmeric extract-mediated biogenic synthesis of Ag@SeO2 magnetic nanoparticles: characterization, optimization, antibacterial and antioxidant activities
Source: RSC Adv. 2024 Feb 27;14(10):7088–111. doi: 10.1039/d4ra00004h (PMC10897959; doi:10.1039/d4ra00004h)
Supplement: RA-014-D4RA00004H-s001 [file RA-014-D4RA00004H-s001.pdf]

Electronic Supplementary Material (ESI) for RSC Advances.
This journal is © The Royal Society of Chemistry 2024

Supplementary data

Turmeric extract-mediated biogenic synthesis of $Ag@SeO_2$ magnetic nanoparticles: Characterization, optimization, antibacterial and antioxidant activities

Abeer A. Ghoniem; Khaled M. Elattar; Fatimah O. Al-Otibi; Ashraf Elsayed; Mohammed S. El-Hersh, Ayman Y. El-Khateeb; Yosra A. Helmy; WesamEldin I. A. Saber

Table S1. The FT-IR absorption bands assignments.

Wavenumber (cm^{-1})	Characteristic Group	Turmeric Extract	$Ag@SeO_2$ bmNPs	Shift (cm^{-1})
**3318**	O-H stretching (phenolic, alcoholic)	✓	✓ (broader)	-
**3296**	O-H stretching (phenolic, alcoholic)	✕	✓ (broader)	-
**2973**	C-H stretching (aliphatic)	✓	✓	-
**2162**	C≡C stretching (unsaturated)	✓	✓ (shifted)	-16
**1652**	C=O stretching (conjugated ketone, aromatic carboxylic acid)	✓	✓ (shifted)	19
**1515**	Amide	✕	✓	-
**1381**	C-H bending (aromatic)	✓	✓	-
**1087, 1043**	C-O stretching (carbohydrate, polysaccharide)	✓	✓	-
**947**	Se-O stretching	✕	✓	-
**878**	C-H in-plane bending (aromatic)	✓	✕	-
**592**	Aromatic skeletal vibrations	✓	✕	-
**486**	Metal-oxygen stretching (Se-O)	✕	✓	-
**430**	O-H out-of-plane bending or C-C/C-N stretching	✓	✕	-

✓ = Peak present; ✕ = Peak absent

Table S2. Particle size distribution estimated from the TEM analysis for Ag@SeO_2 bmNPs.

**Particle size (nm)**	**Number of particles**
10-20	15
21-30	10
31-40	5
41-50	3
51-60	2
> 60	1

Table S3. The results of the XRD pattern of Ag@SeO_2 bmNPs.

Pos. [°2Th.]	Height [cts]	FWHM [°2Th.]	d-spacing [Å]	Rel. Int. [%]	Tip width [°2Th.]	Matched by
28.0653	17.48	0.3149	3.17945	31.77	0.3779	
29.6122	17.19	0.2362	3.01679	31.24	0.2834	00-049-1770
31.8716	55.02	0.3149	2.80790	100.00	0.3779	00-052-0059
34.4904	11.39	0.9446	2.60046	20.70	1.1336	
38.0638	48.52	0.6298	2.36415	88.18	0.7557	01-075-0837; 00-052-0059
44.0496	11.41	0.6298	2.05579	20.73	0.7557	01-075-0837; 00-049-1770
51.9532	11.52	0.7872	1.76011	20.93	0.9446	00-049-1770
54.3324	9.43	0.6298	1.68853	17.14	0.7557	00-049-1770
64.3039	19.79	0.3936	1.44868	35.96	0.4723	01-075-0837
77.3277	12.59	0.3840	1.23297	22.88	0.4608	01-075-0837

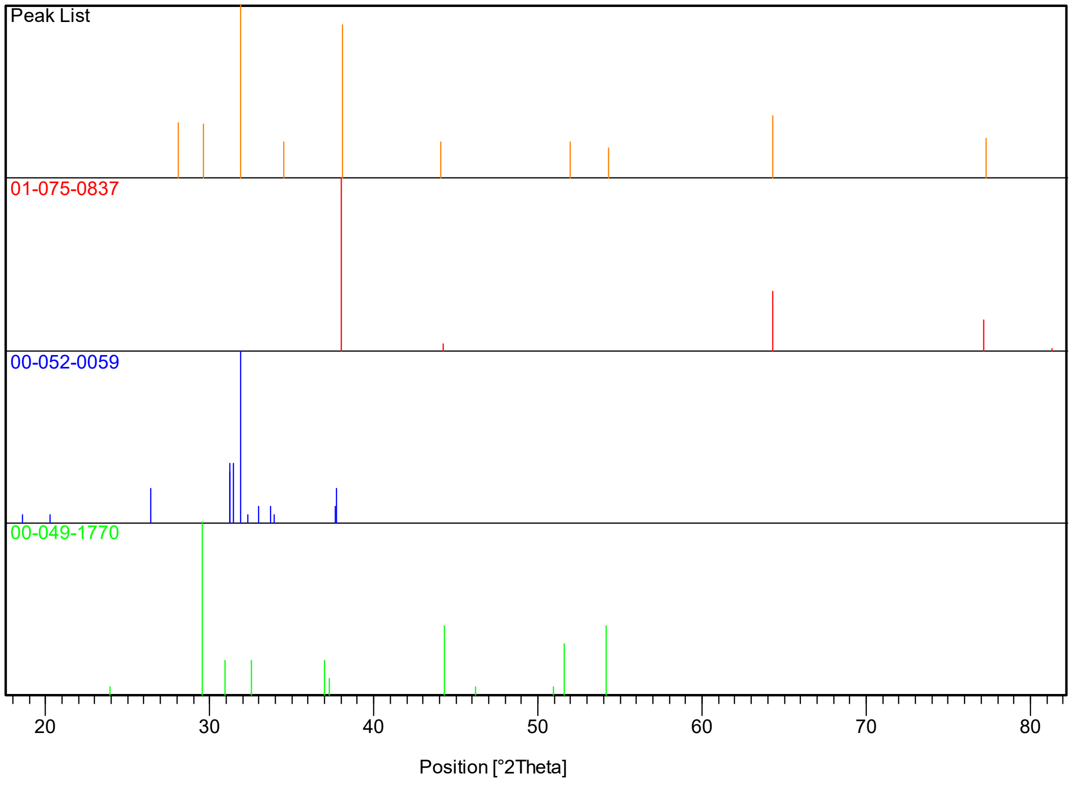

Figure S1. XRD analysis pattern of Ag@SeO_2 bmNPs.

Anchor Scan Parameters:

Dataset Name	Ag-SeO_2 Curcuma
File name	E:\X'Pert Data\2023\10\Dr khaled elatar\Ag-SeO_2 Curcuma\Ag-SeO_2 Curcuma.xrdml
Comment	Configuration=Stage Flat Samples, Owner=r, Creation date=2/28/2009 2:36:26 PM
	Goniometer=PW3050/60 (Theta/Theta); Minimum step size 2Theta:0.001; Minimum step size Omega:0.001
	Sample stage=PW3071/xx Bracket
	Diffractometer system=XPERT-PRO
	Measurement program=program5, Owner=r, Creation date=12/12/2004 11:02:06 AM
Measurement Date / Time	10/2/2023 4:54:03 PM
Operator	cwi
Raw Data Origin	XRD measurement (*.XRDML)
Scan Axis	Gonio
Start Position [°2Th.]	5.0100
End Position [°2Th.]	89.9900
Step Size [°2Th.]	0.0200
Scan Step Time [s]	0.9500
Scan Type	Continuous
Offset [°2Th.]	0.0000
Divergence Slit Type	Fixed
Divergence Slit Size [°]	0.4785
Specimen Length [mm]	10.00
Receiving Slit Size [mm]	0.1000
Measurement Temperature [°C]	25.00
Anode Material	Cu
K-Alpha1 [Å]	1.54060
K-Alpha2 [Å]	1.54443
K-Beta [Å]	1.39225
K-A2 / K-A1 Ratio	0.50000
Generator Settings	30 mA, 40 kV
Diffractometer Type	0000000000005545
Diffractometer Number	0
Goniometer Radius [mm]	240.00
Dist. Focus-Diverg. Slit [mm]	91.00
Incident Beam Monochromator	No
Spinning	No

Table S4. Phytochemical contents of the tested samples.

Sample	Phytochemicals	
	Phenolic content (mg GAE/g)	Flavonoid content (mg CE/g)
**Turmeric extract**	194.038±2.8	170.606±0.59
**Ag@SeO_2 bmNPs**	64.712±1.3	33.362±0.74

GAE: Gallic acid equivalents.

CE: Catechin equivalents.

* The values expressed the mean value ± Standard deviation (SD).

* Values represent individual triplicate measurements.

ABTS Antioxidant Activity

Table S5. The results of antioxidant activity by ABTS assay.

Concentration (µg/mL)	% Inhibition		
	Ascorbic acid	Turmeric extract	Ag@SeO_2 bmNPs
10	24.2	8.5	18.9
20	37.4	19.6	26.1
40	54.9	34.5	31.1
60	66.7	41.5	36.2
80	78.5	50.3	43.0
100	94.3	62.0	65.6
IC_{50} (µg/mL)	29.47±0.17	73.42±0.35	84.48±0.41

* The IC_{50} (µg/mL) values expressed the mean value ± Standard deviation (SD).

* The results represent individual triplicate measurements.

DPPH Antioxidant Activity

Table S6. The antioxidant results (% remaining DPPH, % scavenging activity, and IC_{50} (µg/mL)) of the investigated plants extracted in water.

Sample	Concentrations (µg/mL)	% Remaining DPPH	% Scavenging activity	IC_{50} (µg/ml)
**Turmeric extract**	80	26.89	73.11	42±0.56
	40	49.18	50.82	
	20	73.61	26.39	
	10	87.87	12.13	
**Ag@SeO_2 bmNPs**	673	15.08	84.92	230±1.20
	336	33.93	66.07	
	168	59.02	40.98	
	84	79.84	20.16	
**Ascorbic acid**	62	15.27	84.73	22±0.84
	31	39.08	60.92	
	16	61.07	38.93	
	8	74.81	25.19	

* The IC_{50} (µg/mL) values expressed the mean value ± Standard deviation (SD).

* The results represent individual triplicate measurements.

Minimum Inhibitory Concentration (MIC)

Bacillus cereus

After 24 h of incubation at 37 °C, turbidity was noticed in test tube 8 whereas in tube 7 (MIC) no turbidity was seen, exhibiting inhibition of bacterial growth.

Table S7. The measured O.D 600 for *Bacillus cereus*.

Test tube no.	Concentration (μg/mL)	O.D600
**1**	21200	0.000
**2**	10600	0.002
**3**	5300	0.001
**4**	2650	0.000
**5**	1325	0.010
**6**	662.5	0.010
**7**	331.25	0.007
**8**	**165.625**	**1.12**
**9**	82.8125	1.14
**Control**	-	1.3

MIC tube: NO 7

Klebsiella Pneumoniae

After 24 h of incubation at 37 °C, turbidity was noticed in test tube 8 whereas in tube 7 (MIC) no turbidity was seen, exhibiting inhibition of bacterial growth.

Table S8. The measured O.D 600 for *Klebsiella Pneumoniae*.

**Test tube no.**	**Concentration (μg/mL)**	**O.D$_{600}$**
**1**	21200	0.000
**2**	10600	0.003
**3**	5300	0.001
**4**	2650	0.002
**5**	1325	0.010
**6**	662.5	0.000
**7**	331.25	0.001
8	**165.625**	**1.1**
**9**	82.8125	1.12
**Control**	-	1.01

MIC tube: NO 7

Escherichia coli

After 24 h of incubation at 37 °C, turbidity was noticed in test tube 9 whereas in tube 8 (MIC) no turbidity was seen, exhibiting inhibition of bacterial growth.

Table S9. The measured O.D 600 for *E. coli*.

Test tube no.	Concentration (µg/mL)	$O.D_{600}$
**1**	21200	0.000
**2**	10600	0.002
**3**	5300	0.001
**4**	2650	0.001
**5**	1325	0.000
**6**	662.5	0.010
**7**	331.25	0.002
**8**	165.625	0.001
**9**	**82.8125**	**0.9**
**Control**	-	1.1

MIC tube: NO 8

Staphylococcus aureus

After 24 h of incubation at 37 °C, turbidity was noticed in test tube 9 whereas in tube 8 (MIC) no turbidity was seen, exhibiting inhibition of bacterial growth.

Table S10. The measured O.D 600 for *Staphylococcus aureus*.

Test tube no.	Concentration (µg/mL)	O.D$_{600}$
**1**	21200	0.000
**2**	10600	0.002
**3**	5300	0.001
**4**	2650	0.000
**5**	1325	0.010
**6**	662.5	0.010
**7**	331.25	0.007
**8**	165.625	0.001
9	**82.8125**	**0.78**
**Control**	-	1.2

MIC tube: NO 8

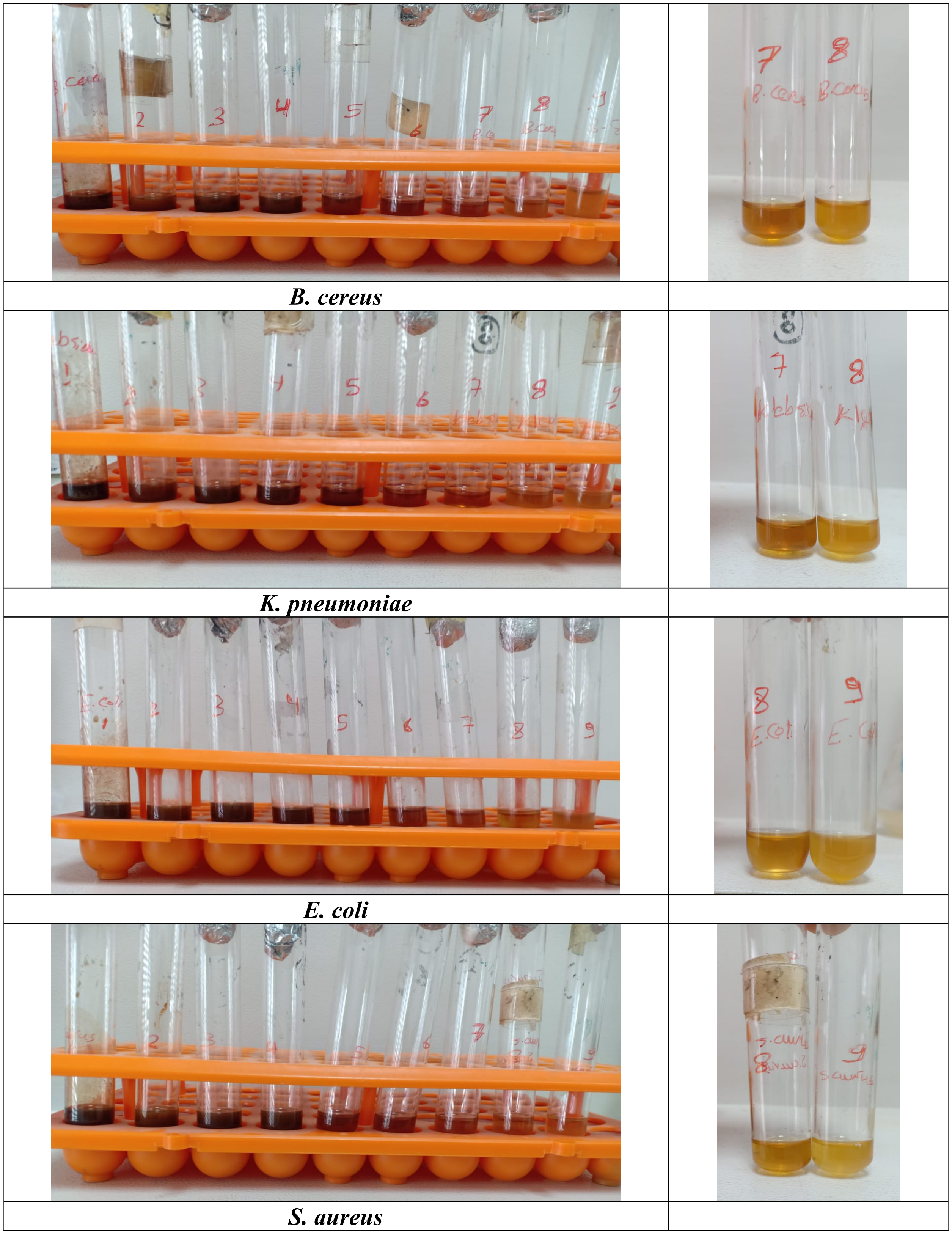

Figure S2. MIC test for Ag@SeO_2 bmNPs against various bacterial species.
